# Nutritional redox reprogramming in cardiometabolic diseases: alpha-lipoic acid, urolithin A, and ergothioneine as modulators of the ferroptosis–mitophagy axis and mitochondrial metabolic remodeling

**DOI:** 10.3389/fnut.2026.1819082

**Published:** 2026-05-15

**Authors:** Wanzhou Yang, Weixiong Jian

**Affiliations:** School of Traditional Chinese Medicine, Hunan University of Chinese Medicine, Changsha, Hunan, China

**Keywords:** alpha-lipoic acid, atherosclerosis, cardiometabolic disorders, ergothioneine, ferroptosis, mitochondrial metabolism, myocardial ischemia, urolithin A

## Abstract

Cardiometabolic disorders, encompassing atherosclerosis, myocardial ischemia, and myocardial infarction, persist as predominant contributors to morbidity and mortality on a global scale. An expanding corpus of research delineates redox imbalance, ferroptosis, compromised mitophagy, and mitochondrial metabolic dysfunction as pivotal determinants of cardiovascular pathophysiology. The paradigm of nutritional redox reprogramming has emerged as a potentially effective approach to modulate these interrelated pathways through the utilization of bioactive dietary compounds. This review emphasizes three novel nutraceutical modulators such as alpha-lipoic acid (ALA), urolithin A (UA), ergothioneine (EGT), and their respective functions in the regulation of ferroptosis, mitochondrial quality control, and cardiac bioenergetics. ALA exhibits multifaceted cardioprotective properties by diminishing oxidative stress, inhibiting lipid peroxidation, enhancing endothelial function, and restoring mitochondrial metabolism in the contexts of atherosclerosis and ischemic injury. UA, a metabolite derived from gut microbiota, primarily promotes mitophagy and mitochondrial biogenesis, thereby augmenting metabolic flexibility and enhancing resistance to ischemic stress. EGT, a thiol antioxidant derived from dietary sources and transported through the OCTN1 transporter, exhibits nascent potential in mitigating oxidative stress and maintaining mitochondrial homeostasis, although the mechanistic insights remain sparse. Collectively, these compounds signify promising candidates for the targeted modulation of redox status in cardiometabolic pathologies. Elucidating their common and unique molecular mechanisms may enhance the formulation of precision nutritional interventions aimed at preventing and mitigating the progression of cardiovascular diseases.

## 1 Introduction

Cardiometabolic disorders, which include Atherosclerosis, myocardial ischemia (MI), type 2 diabetes mellitus (T2DM), obesity, and metabolic syndrome, continue to serve as the predominant contributors to global morbidity and mortality, representing the majority of cardiovascular fatalities on a worldwide scale (1). Notwithstanding significant advancements in lipid-lowering interventions, antithrombotic therapies, glucose-lowering pharmaceuticals, and revascularization techniques, residual cardiovascular risk remains evident even among patients receiving optimal treatment. This enduring challenge underscores a critical drawback of existing paradigms: the majority of therapeutic interventions focus on downstream clinical outcomes rather than addressing the upstream cellular and metabolic determinants. A growing body of evidence pinpoints redox imbalance and mitochondrial dysfunction as pivotal, integrative mechanisms that connect metabolic dysregulation with vascular inflammation, plaque instability, endothelial dysfunction, and ischemia–reperfusion injury (2, 3). Reactive oxygen species (ROS) are increasingly recognized not merely as deleterious byproducts of oxidative metabolism; instead, they are acknowledged as pivotal signaling molecules operating within meticulously regulated parameters.

In the context of cardiometabolic disease, chronic excess of nutrients, hyperglycemia, dyslipidemia, and systemic inflammation alter the redox landscape, leading to persistent oxidative stress. This maladaptive condition compromises nitric oxide bioavailability, induces oxidation of low-density lipoprotein (LDL), activates redox-sensitive transcription factors such as NF-κB, and fosters vascular smooth muscle proliferation and foam cell formation (2). Mitochondria serve as both the primary origin and target of excessive ROS. Damage to mitochondrial DNA, reduced efficiency of the electron transport chain, changes in substrate utilization, and impaired mitochondrial quality control cumulatively exacerbate oxidative stress in a self-reinforcing cycle that accelerates cardiometabolic disease progression (3, 4).

In this framework, ferroptosis has surfaced as a crucial mechanism underlying endothelial damage, cardiomyocyte mortality during ischemia–reperfusion events, and the susceptibility of atherosclerotic plaques (5, 6). Simultaneously, impaired mitophagy disrupts mitochondrial turnover, allowing for the accumulation of dysfunctional, ROS-generating mitochondria that predispose cells to ferroptosis and apoptosis. The convergence of ferroptosis, mitophagy, and mitochondrial metabolic reprogramming delineates a significant yet inadequately integrated axis within the realm of cardiometabolic research. The notion of “nutritional redox reprogramming” transcends traditional antioxidant supplementation paradigms.

Conventional antioxidant investigations employing vitamins C and E have predominantly been unsuccessful in consistently evidencing cardiovascular advantages, partly due to the indiscriminate scavenging of ROS, which interferes with physiological redox signaling and fails to rectify underlying mitochondrial dysfunction (7). Conversely, nutritional redox reprogramming pertains to the precise modulation of redox-sensitive signaling cascades, mitochondrial biogenesis, iron homeostasis, lipid peroxidation pathways, and endogenous antioxidant mechanisms via bioactive dietary compounds that function as hormetic regulators rather than mere passive radical scavengers.

This framework is congruent with the current comprehension of metabolic adaptability, immunometabolism, and precision nutrition. In the context of this evolving paradigm, alpha-lipoic acid (ALA), urolithin A (UA), and ergothioneine (EGT) emerge as mechanistically convergent yet biologically disparate candidates. ALA serves as a mitochondrial cofactor endowed with redox-cycling capabilities, which modulates critical pathways such as AMPK, Nrf2, and insulin signaling, while concurrently facilitating the regeneration of endogenous antioxidants, including glutathione (8). In contrast to traditional antioxidants, ALA exerts influence over mitochondrial bioenergetics and has the potential to mitigate lipid peroxidation and iron-mediated oxidative damage, thereby implicating it in the modulation of ferroptosis. Nonetheless, the clinical application of ALA has been constrained by variability in dosing regimens, bioavailability, and the criteria for patient selection.

UA, a metabolite derived from ellagitannins through the action of gut microbiota, has garnered significant scholarly interest for its role as a robust inducer of mitophagy and mitochondrial biogenesis (9). By enhancing the mechanisms of mitochondrial quality control, UA effectively addresses a fundamental defect observed in cardiometabolic diseases: the persistent presence of dysfunctional mitochondria that exacerbate oxidative stress.

Recent evidence indicates that enhanced mitophagy may indirectly mitigate ferroptotic susceptibility by restricting the accumulation of lipid ROS and reinstating metabolic flexibility. Nonetheless, the majority of existing research has predominantly concentrated on the domains of aging and skeletal muscle, while there remains a relative paucity of integrative analyses in the contexts of vascular and ischemic heart conditions. EGT, a thiol/thione derived from dietary sources and transported into cellular compartments by the OCTN1 transporter, exhibits accumulation within mitochondria and regions characterized by oxidative injury (10). The distinctive redox chemistry of EGT endows it with cytoprotective attributes that safeguard against lipid peroxidation and oxidative damage induced by iron, thereby establishing it as a potential nutrient that modulates ferroptosis. Although observational studies indicate a correlation between elevated EGT concentrations and diminished cardiometabolic risk, there exists a deficiency in mechanistic understanding concerning its influence on mitochondrial metabolism and mitophagy within the contexts of atherosclerosis and MI.

Despite the existence of distinct review articles that have scrutinized oxidative stress in the context of cardiovascular disease, ferroptosis in myocardial injury, mitophagy in metabolic disorders, and specific compounds such as ALA and UA, the body of literature remains considerably disjointed. Previous reviews frequently adopt reductionist paradigms, concentrating solely on antioxidant capabilities or isolating a singular pathway without amalgamating mitochondrial metabolism and nutrient-mediated reprogramming. Furthermore, a scarce number of analyses rigorously explore the reasons underlying the ineffectiveness of broad-spectrum antioxidant approaches, while emphasizing that pathway-specific, mitochondria-targeted interventions may yield more significant outcomes. To the best of our knowledge, no prior review has methodically synthesized ALA, UA, and EGT within a cohesive framework of nutritional redox reprogramming that concurrently examines ferroptosis, mitophagy, and mitochondrial metabolic remodeling in the contexts of atherosclerosis and MI.

A rigorous evaluation of the current body of evidence uncovers numerous deficiencies. Firstly, a significant proportion of preclinical investigations depend on supraphysiological concentrations of bioactive compounds, thus constraining their translational applicability. Secondly, the markers indicative of ferroptosis are variably defined, which complicates the comparability of findings across different studies. Thirdly, mitochondrial functional parameters are frequently deduced indirectly rather than being assessed in a comprehensive manner. Fourthly, the variability among individuals has seldom been integrated into the design of cardiometabolic trials. Rectifying these shortcomings is imperative for transitioning from merely descriptive antioxidant frameworks to mechanism-oriented, precision cardiometabolic strategies. From both the extensive body of scientific literature and empirical observations within the realm of translational redox biology, several salient conclusions can be drawn. Cardiometabolic diseases fundamentally represent disruptions in maladaptive metabolic and redox signaling pathways, rather than mere conditions characterized by lipid accumulation. The deliberate targeting of ferroptosis, without concurrently restoring the integrity of mitochondrial quality control, is improbable to yield sustained therapeutic benefits. Similarly, the induction of mitophagy in the absence of regulatory mechanisms for iron and lipid peroxidation may prove to be inadequate. Nutritional redox reprogramming proposes a comprehensive systems-level strategy wherein meticulously chosen bioactive compounds influence intrinsic defense mechanisms, mitochondrial turnover, and metabolic adaptability.

ALA, UA, and EGT collectively embody a novel class of mitochondria-targeted nutraceuticals that possess the potential to connect molecular pathways with clinical cardiological practice. Nonetheless, the execution of rigorous mechanistic investigations, biomarker-driven clinical trials, and personalized therapeutic approaches is essential prior to the comprehensive integration of these compounds into preventive or therapeutic paradigms for atherosclerosis and MI.

## 2 Chemical structure and key characteristics of ALA, UA, and EGT

ALA is an organosulfur compound naturally synthesized in mitochondria. Chemically, ALA consists of an eight-carbon fatty acid backbone (octanoic acid) covalently modified with a five-membered 1,2-dithiolane ring, which serves as a cyclic disulfide moiety (11). This dithiolane structure is key to ALA’s ability to undergo reversible redox cycling between the oxidized form (ALA) and the biologically active reduced form, dihydrolipoic acid (DHLA). The ALA/DHLA redox couple is one of the most potent biological redox systems identified in mammalian cells (11). While ALA, UA, and EGT share roles in combating oxidative stress in cardiometabolic disease, their chemical structures dictate fundamentally different mechanisms of action.

ALA is characterized by its 1,2-dithiolane ring integrated into a fatty acid backbone. This structure enables a highly efficient, reversible redox cycling between the oxidized form (ALA) and its reduced dithiol form, Dihydrolipoic Acid (DHLA). This thiol-based pair functions as a powerful redox buffer, mimicking the chemical utility of the GSH/GSSG system to maintain the reducing environment within mitochondria and the cytosol. ALA’s amphipathic nature facilitates its access to the mitochondrial matrix, where it supports lipoamide-dependent enzyme function.

EGT is a unique, zwitterionic thiourea derivative of histidine. Its chemical hallmark is the presence of a stable, antioxidant-resistant thione sulfur ( C=S ) within its imidazole ring. Like ALA, EGT operates as a redox cycler, taking up electrons to form a thiol species and participating in robust thiol/thione-based redox buffering pathways that protect cellular components from irreversible damage, mediated by its specific transporter (OCTN1).

In contrast, UA is a phenolic dibenzopyranone. Lacking a thiol group capable of forming a reversible disulfide bond, UA acts primarily as a monostable antioxidant via its hydroxyl group, scavenging radicals similarly to vitamins C and E. Furthermore, UA’s biological impact is heavily weighted toward signaling, particularly the activation of mitochondrial quality control pathways like mitophagy.

In mammalian tissues, ALA functions as an essential enzyme-bound cofactor for mitochondrial α-ketoacid dehydrogenase complexes, including pyruvate dehydrogenase and α-ketoglutarate dehydrogenase (12). Through its covalent attachment to lysine residues of E2 subunits, ALA participates in acyl-transfer reactions and electron shuttling, serving as a central mediator of mitochondrial oxidative decarboxylation (13).

ALA and DHLA also exert broad antioxidant and anti-inflammatory activity. DHLA, due to its increased nucleophilicity, regenerates glutathione (GSH), vitamin C, vitamin E, and coenzyme Q10, thereby strengthening endogenous antioxidant systems (13). ALA additionally activates Nrf2, the principal transcription factor governing antioxidant gene expression, promoting the upregulation of heme oxygenase-1 (HO-1), NAD(P)H quinone dehydrogenase 1 (NQO1), and glutamate-cysteine ligase. Through interactions with AMPK and insulin-sensitive pathways, ALA also improves metabolic signaling and glucose utilization (13).

Structurally driven properties of ALA, particularly its disulfide ring and amphipathic nature, allow rapid cellular uptake and mitochondrial accumulation. These features underlie its documented ability to reduce ROS, suppress lipid peroxidation, modulate iron-dependent oxidative injury, and attenuate ferroptotic signaling in cardiomyocytes and endothelial cells (14).

**Figure 1:**
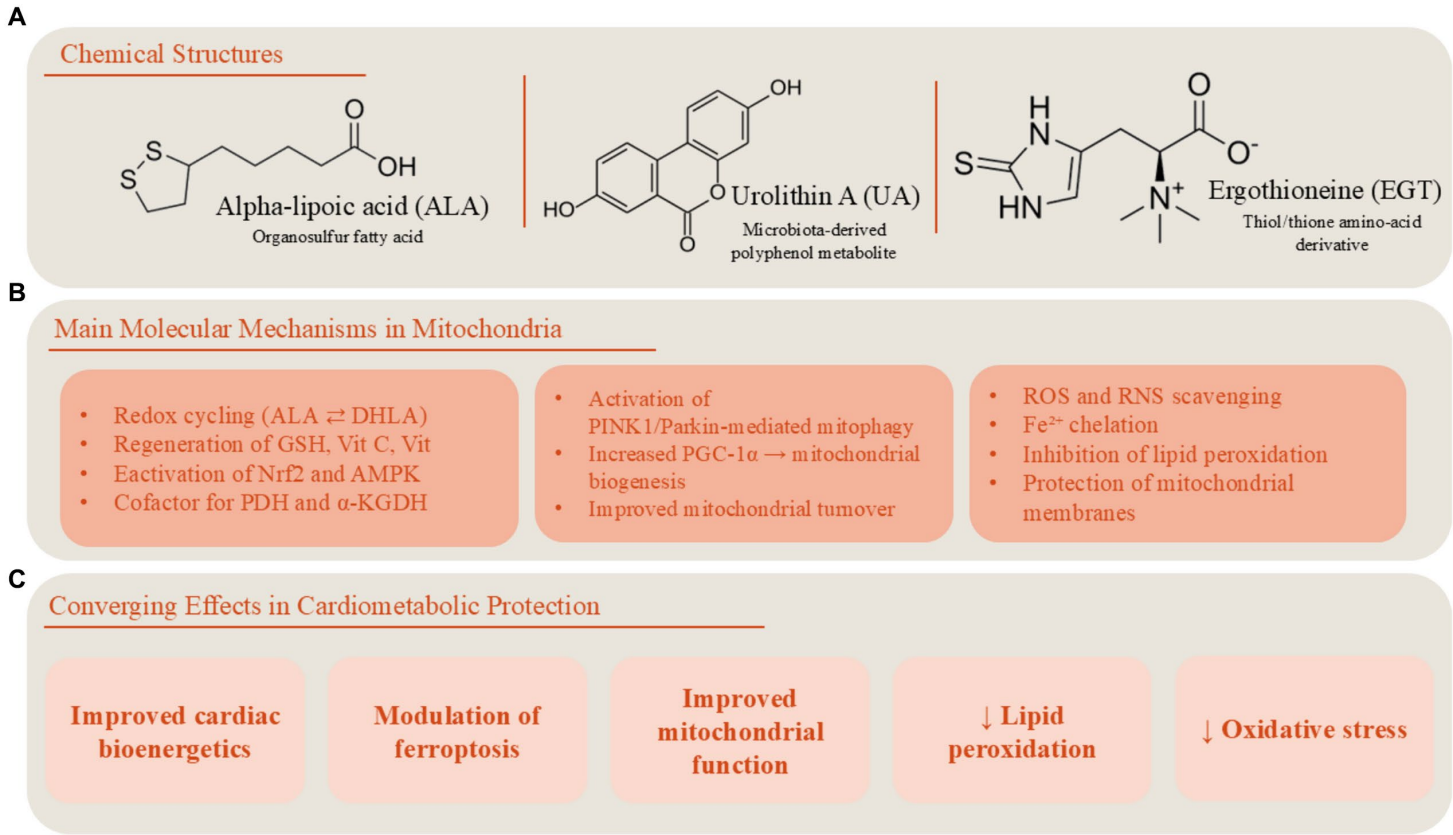
Chemical structures and mitochondrial mechanisms of ALA, urolithin A, and ergothioneine. Panel **(A)** illustrates the chemical structures of alpha-lipoic acid (ALA), urolithin A (UA), and ergothioneine (EGT), highlighting key functional groups that underlie their biochemical activity: the 1,2-dithiolane ring of ALA, the dibenzo-α-pyrone core of UA, and the imidazole-based thiol/thione structure of EGT. Panel **(B)** depicts the principal mitochondrial mechanisms engaged by each compound. ALA participates in redox cycling (ALA ⇄ DHLA), supports antioxidant regeneration, and serves as a cofactor for key mitochondrial dehydrogenases. UA enhances mitochondrial quality control through stimulation of PINK1/Parkin-dependent mitophagy and promotes biogenesis via PGC-1α activation. EGT functions as a potent antioxidant that scavenges reactive oxygen and nitrogen species, chelates redox-active metals, and protects mitochondrial membranes from lipid peroxidation. Panel **(C)** summarizes the convergent physiological outcomes of these pathways, including reduced oxidative stress, improved mitochondrial function and bioenergetics, and mitigation of lipid peroxidation and ferroptotic vulnerability. Together, the figure connects the structural features of these compounds to their shared and complementary roles in supporting mitochondrial health.

ALA exerts its distinctive antioxidant and cytoprotective properties primarily through its role in mitochondrial redox regulation and metabolic enzyme function (Figure 1) (12). Structurally, ALA contains a redox-active disulfide ring that can be reversibly reduced to dihydrolipoic acid (DHLA), forming a powerful intracellular redox couple. This ALA/DHLA system enables direct scavenging of reactive oxygen and nitrogen species while also regenerating other endogenous antioxidants, including glutathione, vitamin C, vitamin E, and coenzyme Q10 (15). In addition to its chemical antioxidant activity, ALA functions as an essential cofactor for mitochondrial α-ketoacid dehydrogenase complexes, including pyruvate dehydrogenase and α-ketoglutarate dehydrogenase, thereby supporting mitochondrial energy metabolism. Experimental studies further show that ALA activates redox-sensitive signaling pathways such as nuclear factor erythroid 2–related factor 2 (Nrf2) and AMP-activated protein kinase (AMPK), promoting the expression of antioxidant enzymes and improving cellular metabolic adaptability under oxidative stress conditions (12, 15).

UA (3,8-dihydroxy-6H-dibenzo[b,d]pyran-6-one) is a gut microbiota–derived metabolite produced from dietary ellagitannins and ellagic acid (16). Structurally, UA belongs to the dibenzo-α-pyrone class of polyphenolic compounds. Its rigid tricyclic aromatic scaffold and hydroxyl substitutions confer moderate lipophilicity and facilitate passive diffusion across biological membranes (17).

Despite lacking strong direct antioxidant capacity compared with classical polyphenols, UA is distinguished by its ability to activate mitochondrial quality-control pathways, most notably mitophagy. In seminal studies, UA was shown to induce PINK1- and Parkin-dependent mitophagy, improving mitochondrial turnover and extending lifespan in *C. elegans*, as well as enhancing muscle performance in rodents (18). These findings have been replicated in human skeletal muscle and cardiomyocyte models, where UA increases mitochondrial biogenesis through PGC-1α signaling (18). UA alleviates the accumulation of dysfunctional mitochondria, decreases mitochondrial ROS, and rebalances mitochondrial dynamics (fusion/fission) under metabolic stress. By reducing the pool of damaged mitochondria, UA indirectly suppresses ROS-driven inflammatory activation, improves ATP generation, and increases metabolic flexibility—functions particularly relevant to cardiometabolic pathology (19). The microbiota-dependent formation of UA is itself influenced by host metabolic state and gut microbial composition, contributing to inter-individual variability in circulating UA levels. Nonetheless, the molecule’s unique structural arrangement and metabolic stability result in high bioavailability compared with its dietary precursors (19).

UA displays a distinct biological profile characterized by its ability to regulate mitochondrial quality control mechanisms. UA improves cellular resilience by stimulating mitophagy, the selective autophagic removal of damaged mitochondria (Figure 1) (20). This process prevents the accumulation of dysfunctional mitochondria that generate excessive reactive oxygen species and impair cellular bioenergetics. Experimental studies demonstrate that UA activates mitophagy pathways involving key regulators such as PINK1 and Parkin and promotes mitochondrial biogenesis, thereby improving mitochondrial turnover and metabolic efficiency (20). Through these mechanisms, UA enhances mitochondrial function, improves oxidative metabolism, and increases resistance to metabolic and oxidative stress, processes that are particularly relevant in aging and cardiometabolic disease (21).

EGT is a naturally occurring, diet-derived sulfur-containing compound that belongs to the class of thiol/thione antioxidants (22). It is synthesized exclusively by certain fungi and microorganisms and subsequently enters the human body primarily through the consumption of foods such as mushrooms and some fermented products (22, 23). Chemically, EGT is a derivative of the amino acid histidine in which the imidazole ring is modified to contain a sulfur atom, forming a stable thiol/thione tautomeric system. This unusual chemical configuration confers distinctive redox properties, allowing EGT to participate in antioxidant reactions while remaining remarkably resistant to irreversible oxidation. As a result, EGT can maintain redox stability under physiological conditions and function as a persistent intracellular antioxidant (22, 23).

A defining feature of EGT biology is its highly selective cellular uptake mediated by the organic cation transporter novel type-1 (OCTN1; also known as SLC22A4). This transporter exhibits a strong specificity for EGT and facilitates its efficient accumulation in tissues exposed to elevated oxidative stress, including the liver, kidneys, immune cells, and the cardiovascular system. Importantly, OCTN1-mediated transport allows EGT to concentrate within mitochondria and other metabolically active cellular compartments where ROS generation is prominent. Such targeted localization suggests that EGT may play a specialized role in protecting mitochondrial structures and functions from oxidative injury (24, 25).

The thiol/thione redox system of EGT enables it to neutralize a wide spectrum of reactive species, including hydroxyl radicals, singlet oxygen, and peroxynitrite. Unlike many conventional antioxidants that become rapidly depleted following oxidation, EGT demonstrates exceptional chemical stability, allowing it to persist within cells and maintain long-lasting antioxidant activity. Through these properties, EGT contributes to the maintenance of cellular redox homeostasis and protects biomolecules such as lipids, proteins, and nucleic acids from oxidative damage (25).

Within mitochondria and other vulnerable cellular compartments, EGT has been shown to safeguard membrane phospholipids against lipid peroxidation and to limit oxidative injury mediated by redox-active metals, particularly iron. By reducing iron-dependent oxidative reactions and inhibiting the propagation of lipid peroxidation chains, EGT may interfere with key molecular events that initiate ferroptosis, a regulated form of cell death characterized by iron-driven phospholipid peroxidation. Consequently, EGT has been proposed as a potential nutritional modulator of ferroptotic signaling pathways, particularly in tissues with high metabolic demand such as the heart and vascular endothelium (24).

Emerging epidemiological and observational studies suggest that higher circulating or tissue concentrations of EGT are associated with reduced markers of oxidative stress, improved metabolic health, and a lower risk of cardiometabolic disorders (26). These associations raise the possibility that EGT may contribute to cardiovascular protection by preserving mitochondrial integrity and limiting oxidative damage in vascular and myocardial tissues. Nevertheless, despite growing interest in EGT as a mitochondrial-targeted antioxidant, the precise molecular mechanisms through which it influences mitochondrial metabolism, mitophagy, and cellular stress responses remain incompletely understood. In particular, experimental evidence elucidating its role in mitochondrial quality control pathways and its potential interactions with ferroptotic or inflammatory signaling in atherosclerosis and myocardial infarction is still limited. Further mechanistic and translational studies are therefore required to clarify the full cardioprotective potential of this unique thiol/thione antioxidant (26, 27).

EGT functions primarily as a cytoprotective antioxidant, capable of scavenging superoxide, hydroxyl radicals, hypochlorous acid, singlet oxygen, and peroxynitrite (28). It also strongly chelates divalent metal ions, including Fe^2+^, thereby reducing the availability of catalytic iron required for Fenton chemistry and ferroptotic lipid peroxidation (28).

Due to its chemical stability and capacity for intracellular concentration, EGT preserves mitochondrial membrane potential, limits cardiomyocyte oxidative damage, and protects against ischemia-related metabolic stress. Although mechanistic studies in cardiometabolic disease are fewer than for ALA and UA, existing evidence points to a role in modulating redox homeostasis, mitochondrial integrity, and inflammatory responses (29).

## 3 Redox reprogramming in cardiometabolic diseases

Redox reprogramming in cardiometabolic disorders signifies a transition from normative redox signaling to a persistently oxidized intracellular milieu that alters transcriptional networks, metabolic pathways, and cellular fate determinations (30). In pathological states such as Atherosclerosis and MI, oxidative stress transcends being a mere consequence of metabolic overload, emerging as a pivotal regulatory element that orchestrates endothelial dysfunction, lipid peroxidation, inflammatory responses, and the demise of cardiomyocytes (31). It is crucial to recognize that redox dysregulation and mitochondrial impairment establish a self-reinforcing loop: excessive mitochondrial ROS compromise respiratory efficacy and mitochondrial DNA stability (32), while impaired mitochondrial turnover exacerbates ROS production. This maladaptive reprogramming incorporates ferroptosis, mitophagy, and metabolic rigidity into a cohesive pathogenic framework that intensifies vascular and myocardial damage.

### 3.1 Oxidative stress and ferroptosis in atherosclerosis and myocardial injury

Oxidative stress has been extensively recognized as a significant factor in the pathogenesis of atherosclerosis, particularly through the oxidation of LDL, the activation of redox-sensitive transcription factors, and the reduction of bioavailability of endothelial nitric oxide (2, 33). Nevertheless, traditional antioxidant frameworks have not yielded consistent clinical advantages, indicating that the role of redox biology in cardiovascular pathology is more intricate than merely an excess of ROS (34). Novel findings have identified ferroptosis as a pivotal mechanism underlying vascular and myocardial damage (5). In atherosclerotic lesions, the accumulation of iron, the presence of membranes rich in polyunsaturated fatty acids, and the reduced activity of glutathione peroxidase 4 establish a conducive environment for the initiation of ferroptotic signaling (35). Endothelial cells subjected to oxidized lipids demonstrate elevated levels of lipid-derived ROS and mitochondrial impairment, while macrophage foam cells exhibit dysregulation of iron homeostasis that may exacerbate plaque instability (36). Experimental investigations have revealed that inhibitors of ferroptosis mitigate endothelial dysfunction and the progression of atherosclerotic lesions, thereby emphasizing its functional significance rather than a mere epiphenomenal correlation (6). In the framework of MI–reperfusion, the sudden reintroduction of oxygen instigates a surge of mitochondrial ROS and iron-mediated lipid peroxidation, thereby rendering cardiomyocytes susceptible to ferroptotic cell death (37). In contrast to apoptosis and necrosis, ferroptosis is characterized by extensive damage to membrane lipids and is intricately associated with mitochondrial metabolic dysfunctions (38). However, notable discrepancies persist within the literature: biomarkers indicative of ferroptosis are defined in a heterogeneous manner, and the interaction between ferroptosis and other forms of cell death remains insufficiently elucidated. An essential deficiency exists in the synthesis of iron metabolism, mitochondrial bioenergetics, and lipid remodeling within a comprehensive systems-level redox paradigm.

### 3.2 Mitophagy and mitochondrial quality control in cardiovascular pathology

Mitochondrial quality control, with a particular emphasis on mitophagy, is critical for sustaining cardiac homeostasis due to the heart’s exceptional energetic requirements. The PINK1/Parkin signaling pathway, among other mechanisms, facilitates the selective elimination of impaired mitochondria, thereby mitigating excessive ROS generation and conserving bioenergetic efficiency (4). In the context of cardiometabolic diseases, compromised mitophagy results in the accumulation of dysfunctional mitochondria, which exacerbates oxidative stress and predisposes cells to ferroptosis and apoptosis. In atherosclerotic blood vessels, impaired mitophagy within endothelial and smooth muscle cells incites inflammatory activation and phenotypic transformation. Likewise, in ischemic myocardial tissue, inadequate mitophagic flux worsens post-ischemic remodeling and contractile impairment (39). Conversely, excessive or unregulated mitophagy may also jeopardize mitochondrial mass and energy production, underscoring the necessity for precise regulation of mitophagy as opposed to indiscriminate activation (40). Current review frequently characterizes mitophagy as a distinct protective mechanism; however, recent evidence indicates the presence of bidirectional crosstalk with ferroptosis. Deficiencies in mitochondrial turnover result in heightened lipid ROS production and disrupt iron–sulfur cluster homeostasis, which may diminish the threshold for ferroptotic cellular demise. In contrast, ferroptotic stress could compromise mitochondrial dynamics and membrane integrity, thereby exacerbating the impairment of mitophagic recognition. Notwithstanding the accumulation of mechanistic insights, translational investigations that connect the modulation of mitophagy to clinical outcomes in cardiovascular health are notably limited, and there is a lack of standardized methodologies for quantifying mitophagic flux within human tissues.

### 3.3 Mitochondrial metabolism and metabolic flexibility in ischemic heart disease

The healthy human heart demonstrates exceptional metabolic adaptability, efficiently transitioning between fatty acid oxidation and glucose metabolism contingent upon substrate availability and energetic requirements. In the context of ischemic heart disease, this adaptability is progressively diminished. Prolonged metabolic stress and redox imbalance alter substrate preference, hinder pyruvate dehydrogenase activity, and decrease the efficacy of oxidative phosphorylation, ultimately resulting in energetic insufficiency (3, 41). During episodes of acute ischemia, diminished oxygen levels inhibit mitochondrial respiration and heighten dependence on anaerobic glycolysis. Following reperfusion, the swift reactivation of the electron transport chain in the presence of accumulated reducing equivalents leads to a surge in ROS (42), which contributes to mitochondrial permeability transition and subsequent cell death. Ongoing mitochondrial dysfunction subsequent to infarction fosters adverse remodeling and exacerbates the progression of heart failure. Importantly, the reprogramming of mitochondrial metabolism exhibits critical intersections with both ferroptosis and mitophagy. Alterations in the flux of the tricarboxylic acid cycle significantly affect the availability of NADPH (43), which serves as a pivotal factor in the regeneration of glutathione and the detoxification of lipid peroxides. Dysregulated metabolism of fatty acids results in modifications to the composition of membrane lipids, potentially heightening vulnerability to peroxidative damage. Concurrently, inadequate mitophagy restricts the replacement of metabolically deficient mitochondria, thereby entrenching cardiomyocytes in a state characterized by oxidative inefficiency. Current research endeavors often examine mitochondrial metabolism, ferroptosis, or mitophagy in a discrete fashion. Nonetheless, the progression of cardiometabolic diseases is indicative of integrated disruptions across these interconnected domains. A perspective grounded in redox reprogramming underscores that mitochondrial dysfunction operates as both a contributor to and a consequence of oxidative stress, positing that the restoration of metabolic flexibility necessitates a comprehensive modulation of iron homeostasis, control of lipid peroxidation, and mitochondrial turnover. This integrative comprehension furnishes the conceptual framework for the strategic targeting of nutrient-derived modulators that have the capacity to concurrently influence redox signaling, mitophagy, and mitochondrial bioenergetics.

## 4 ALA: a multifunctional redox modulator in cardiovascular disease

ALA is significant due to its positioning of this molecule beyond the traditional classification of antioxidants, thereby establishing it as a systems-level redox modulator pertinent to cardiovascular pathology. In the context of cardiometabolic diseases, the persistent challenges of oxidative stress, ferroptotic signaling, and mitochondrial dysfunction remain insufficiently addressed by conventional therapeutic interventions. ALA possesses a unique capacity to target mitochondrial metabolism, restore endogenous antioxidants, modulate the Nrf2 and AMPK signaling pathways, and may exert effects on iron-dependent lipid peroxidation. The integration of these mechanisms elucidates the potential of ALA to reprogram maladaptive redox networks associated with atherosclerosis and MI, thus facilitating a connection between mechanistic understanding and the applications of translational and precision cardiology.

### 4.1 ALA and atherosclerosis progression

Autosomal dominant polycystic kidney disease (ADPKD) is distinguished by the presence of bilateral renal cysts, the early onset of hypertension, endothelial dysfunction, systemic inflammation, and expedited atherosclerosis, thereby augmenting cardiovascular risk and facilitating the progression to end-stage renal disease. A controlled, prospective investigation assessed a cohort of 59 ADPKD patients diagnosed with chronic kidney disease at stages G2/G3, among whom 33 individuals received a daily dosage of 1.6 g of ALA for a duration of 6 months. ALA was found to significantly enhance various metabolic parameters (including glucose, insulin, HOMA-IR, and uric acid), improve endothelial function (evidenced by an increase in flow-mediated dilation and a reduction in the renal resistive index), stabilize acid–base balance, and mitigate oxidative stress (indicated by a decrease in NADPH oxidase 2), in addition to boosting psychocognitive outcomes (measured by the Mini-Mental State Examination, Hamilton Depression Rating Scale, and Beck Depression Inventory-II). No statistically significant alterations were recorded in intima-media thickness, ankle-brachial index, interleukin-6 (IL-6), interleukin-1 beta (IL-1β), or tumor necrosis factor-alpha (TNF-α). ALA demonstrates a profile of safety as an antioxidant and anti-inflammatory agent, exhibiting cardiovascular and cognitive advantages in the early stages of ADPKD (44) (Table 1). Postmenopausal females experience an augmented susceptibility to atherosclerosis as a consequence of the decline in estradiol levels. Although hormone replacement therapy demonstrates immediate cardiovascular advantages, it concomitantly poses elevated risks for stroke and malignancies, underscoring the imperative for safer therapeutic alternatives. Shen et al. assessed the efficacy of ALA, a robust natural antioxidant, in mitigating atherosclerosis utilizing Ldlr^−/−^ murine models and human aortic endothelial cells (HAECs). ALA markedly diminished atherosclerotic lesions precipitated by ovariectomy and a high-fat diet, reinstated the expression of estrogen receptors (ERα, ERβ), attenuated monocyte adhesion, inhibited apoptosis, decreased ROS through the downregulation of Nox4 and p22phox, and obstructed NF-κB activation in HAECs. ALA may represent a promising therapeutic strategy for the management of postmenopausal atherosclerosis (45).

**Table 1:** Cardiovascular and anti-atherosclerotic effects of α-lipoic acid: Mechanisms, cellular targets, and animal/human models.

Experimental model	Molecular targets/pathway	Effects on oxidative stress, inflammation and metabolism	Effects on cardiovascular/vascular function	References
59 patients with ADPKD, CKD stage G2/G3; 33 treated with 1.6 g/d ALA for 6 months	NADPH oxidase 2; inflammatory cytokines (IL-6, IL-1β, TNF-α)	↓ C-reactive protein; ↓ NADPH oxidase 2; no significant change in IL-6, IL-1β, TNF-α	↑ flow-mediated dilation (FMD); ↓ renal resistive index (RRI); no change in IMT or ABI	(44)
Ldlr^−^/^−^ mice (ovariectomized, high-fat diet); human aortic endothelial cells (HAECs) exposed to H2O2	ROS-generating enzymes Nox4, p22phox; NF-κB; estrogen receptors (ERα, ERβ)	↓ intracellular ROS; ↓ NF-κB activation; ↓ monocyte adhesion	↓ atherosclerotic lesion formation; reduced apoptosis in HAECs; restored ERα/ERβ expression	(45)
Human vascular smooth muscle cells treated with TNFα ± 2 mM ALA	Proteins: PAI-2, LKB-interacting protein, osteoblast-specific factor 2, glucosidase II, CDK3, endoplasmin, glutathione synthetase; Rho GDP dissociation inhibitor α; gene expression profiling	ALA reversed TNFα-induced upregulation of pro-inflammatory and oxidative stress-related proteins; restored expression of protective proteins	Implications for reduced VSMC dysfunction and atherosclerosis progression	(46)
Hypercholesterolemic New Zealand White rabbits; 1% cholesterol diet ± 4.2 mg/kg ALA for 10 weeks	Lipid peroxidation (MDA)	↓ plasma total cholesterol and LDL; ↓ MDA formation	↓ aortic intimal lesion formation; reduced atheromatous plaques	(47)
C57BL/6 mice fed hypercholesterolemic diet + LPS; *in vitro* VSMCs	Ras-MEK1/2-ERK1/2 signaling; platelet-derived growth factor (PDGF)	↓ serum cholesterol; ↓ inflammatory cytokines	↓ atherosclerotic lesions; inhibited VSMC proliferation and migration	(48)
ApoE^−^/^−^ mice made diabetic with STZ; high-fat/low-cholesterol diet ± 1.65 g/kg ALA	Oxidative stress pathways; pancreatic β-cell protection	↓ oxidative stress; ↓ plasma glucose	↓ atherosclerotic lesion formation; prevented plasma cholesterol increase	(49)
Hypercholesterolemic New Zealand White rabbits; 1% cholesterol diet ± 4.2 mg/kg ALA for 10 weeks	Lipid peroxidation (MDA)	↓ plasma total cholesterol (TCHOL) and LDL; ↓ MDA formation	↓ aortic intimal lesion formation; reduced atheromatous plaques	(50)
ApoE^−/−^ and apoE/LDLR^−/−^ mice fed Western-type diet ± 0.2% LA for 10 weeks	Endothelial adhesion molecules, proinflammatory cytokines, macrophage accumulation	↓ serum triglycerides; ↓ proinflammatory cytokines	↓ aortic lesion formation in sinus, arch, and thoracic aorta	(51)
Rats fed high-fat diet ± flaxseed oil + 8 g/kg ALA for 10 weeks	Antioxidant enzymes (SOD, CAT, GPx), GSH, IL-6, CRP	↑ antioxidant defense; ↓ lipid peroxidation, ↓ TG, TC, LDL; ↑ HDL/LDL ratio	Not directly measured; improvements inferred from lipid and inflammatory markers	(52)
Atherosclerotic mice; inflammatory macrophage model	Oxidative stress, inflammatory cytokines, macrophage infiltration	↑ Antioxidant activity; ↓ ROS; ↓ proinflammatory cytokines	↓ atherosclerotic lesion development; antiatherosclerotic effect	(53)
Rat vascular smooth muscle cells (VSMCs)	NF-κB-dependent fractalkine expression	↓ TNF-α-induced fractalkine	Prevents VSMC-mediated vascular inflammation	(54)
Type 2 diabetic rats	ICAM-1 expression	↓ ICAM-1 expression	Reduces endothelial activation	(55)
Streptozotocin-induced T2DM Sprague–Dawley rats	Oxidative stress, hyperlipidemia, insulin resistance	↓ glucose, ↓ lipid profile, ↓ IR, ↑ antioxidant enzyme GH-Px	Prevented coronary artery atherosclerotic plaque formation	(56)

ADPKD, autosomal dominant polycystic kidney disease; CKD, chronic kidney disease; KDIGO, Kidney Disease Improving Global Outcomes; FMD, flow-mediated dilation; RRI, renal resistive index; IMT, intima-media thickness; ABI, ankle-brachial index; MMSE, Mini-Mental State Examination; HAM-D, Hamilton Depression Rating Scale; BDI-II, Beck Depression Inventory-II; ROS, reactive oxygen species; HAECs, human aortic endothelial cells; NF-κB, nuclear factor kappa-light-chain-enhancer of activated B cells; TNF-α, tumor necrosis factor-alpha; VSMCs, vascular smooth muscle cells; HCD, high cholesterol diet; LDL, low-density lipoprotein; TCHOL, total cholesterol; TG, triglyceride; ALA, alpha-lipoic acid; STZ, streptozotocin; IR, insulin resistance; LA NPs, lipoic acid nanoparticles; ICAM-1, intercellular adhesion molecule-1; GH-Px, glutathione peroxidase.

The objective of a study was to determine the effects of TNFα (10 ng/mL) on vascular smooth muscle cells (VSMCs) over a duration of 24 h, both in the presence and absence of ALA (2 mM), employing two-dimensional gel electrophoresis and DNA microarray methodologies. Proteins up-regulated by TNFα, such as plasminogen activator inhibitor-2 and cyclin-dependent kinase 3, exhibited down-regulation upon treatment with ALA, whereas proteins down-regulated by TNFα, including keratin 19 and Rho GDP dissociation inhibitor alpha, demonstrated restoration in the presence of ALA. The analysis of gene expression corroborated these alterations to some extent, thereby elucidating the underlying mechanisms of atherosclerosis and the protective function of ALA (46). Amom et al. aimed to examine the protective properties of ALA in hypercholesterolemic New Zealand White rabbits. Eighteen rabbits were categorized into three distinct groups: normal control (N), high-cholesterol diet (HCD), and HCD + ALA groups (4.2 mg/kg body weight) over a period of 10 weeks. Blood samples were procured for the evaluation of lipid profiles and malondialdehyde (MDA) levels, while aortas were subjected to analysis for intimal lesions. ALA supplementation resulted in a significant decrease in total cholesterol, LDL, and MDA concentrations when compared to the HCD group. Histological assessments revealed a reduction in the formation of atheromatous plaques. These findings suggested that ALA exhibits both lipid-lowering and anti-atherosclerotic properties, thereby mitigating oxidative stress and the progression of athero-lesions in hypercholesterolemic rabbits (47). The infectious burden is implicated in the pathogenesis of atherosclerosis through common inflammatory pathways; however, interventions aimed at mitigating atherosclerosis under these circumstances remain inadequately investigated. Lee et al. demonstrated the effects of ALA in C57BL/6 mice subjected to a hypercholesterolemic diet over a duration of 12 weeks, with the administration of lipopolysaccharide in the first week to simulate infectious stimuli. ALA administration resulted in a significant reduction in serum cholesterol levels, inflammatory cytokines, and atherosclerotic lesions, while simultaneously enhancing atherosclerotic biomarkers. From a mechanistic perspective, ALA was found to impede the proliferation and migration of VSMCs stimulated by platelet-derived growth factor through the Ras-MEK1/2-ERK1/2 signaling pathway (48).

Hyperglycemia-induced oxidative stress plays a pivotal role in the progression of atherosclerosis and the onset of cardiovascular complications associated with diabetes. An investigation assessed the influence of ALA (1.65 g/kg) in streptozotocin-induced diabetic apoE^(−/−)^ murine models and their nondiabetic counterparts subjected to a high-fat dietary regimen. Following a duration of 20 weeks, ALA exhibited a substantial reduction in markers of oxidative stress, inhibited elevations in plasma total cholesterol levels, curtailed the development of atherosclerotic lesions, and alleviated health decline associated with diabetes. Furthermore, ALA resulted in a decrease in plasma glucose concentrations and facilitated the recovery of pancreatic insulin-producing cells, thereby suggesting protective effects on beta-cell functionality (49). The aim of a study was to assess the protective properties of ALA (4.2 mg/kg/day) in New Zealand White rabbits subjected to a 1% HCD over a duration of 10 weeks. Blood specimens were obtained for the evaluation of lipid profiles and MDA quantification, while aortic tissues were scrutinized for the presence of intimal lesions. The administration of ALA resulted in a statistically significant decline in MDA, total cholesterol, and LDL concentrations in comparison to the high-cholesterol control group. Histopathological examination indicated a reduction in atheromatous plaque development within the ALA-treated cohort (50).

Zhang et al. assessed the effects of ALA in apoE^−/−^ and apoE/LDL receptor-deficient murine models subjected to either a Western-type diet or a standard chow diet, with or without the inclusion of 0.2% ALA over a duration of 10 weeks. The administration of ALA resulted in a significant reduction of atherosclerotic lesions, approximately 20% in the aortic sinus and between 40 and 55% in both the aortic arch and thoracic aorta. These observed outcomes were correlated with a reduction of around 40% in body weight gain, diminished serum triglycerides and VLDL levels, decreased accumulation of aortic macrophages, lowered expression of adhesion molecules, and a reduction in proinflammatory cytokines. The anti-obesity, antihypertriglyceridemic, and anti-inflammatory properties of ALA are likely responsible for its antiatherogenic effects, indicating its potential role as a preventive and therapeutic adjunct in the management of atherosclerotic vascular disease (51). The objective of a research was to determine the effects of combined flaxseed oil (FO) and ALA supplementation in rats fed a high-fat diet for 10 weeks. ALA was dissolved in FO at 8 g/kg, with varying proportions in the diet. FO+ALA supplementation significantly enhanced plasma antioxidant defenses, increasing SOD, CAT, GPx activities, and GSH levels, while reducing lipid peroxidation. It improved lipid profiles by lowering TG, TC, and LDL-C and increasing the HDL-C/LDL-C ratio. FO+ALA also reduced inflammatory markers IL-6 and CRP (52). Another study engineered LA nanoparticles (LA NPs) through a self-assembly process, utilizing the inherent antioxidant and anti-inflammatory characteristics of LA to enhance bioavailability and mitigate the potential toxicity associated with traditional nanomedicines. In models of inflammatory macrophages, LA NPs demonstrated superior anti-inflammatory efficacy compared to free LA. In a murine model of atherosclerosis, LA NPs diminished plaque development, alleviated oxidative stress, restricted macrophage infiltration, and reduced levels of inflammatory cytokines, as substantiated by ultrasound and immunofluorescence evaluations (53). Another study aimed to elucidate whether ALA exerts an inhibitory effect on the expression of fractalkine that is stimulated by TNF-alpha in rat VSMCs. Analyses conducted using Northern and Western blot techniques revealed that TNF-alpha significantly enhanced fractalkine expression, which was notably attenuated by ALA. Gel shift assays suggested that the inhibitory mechanism of ALA operates, at least in part, through the NF-κB signaling pathway (54).

In a study, it was assessed the impact of ALA (60 mg/kg/day over a period of 21 days) on the expression levels of ICAM-1 within T2DM rat models. A total of 18 male rats were systematically allocated into three distinct groups: control, T2DM, and T2DM + ALA. The expression of ICAM-1 in the abdominal aorta was evaluated through immunohistochemical techniques and subsequently quantified utilizing image analytical methods. T2DM rats demonstrated a statistically significant elevation in both the area and intensity of ICAM-1 expression when compared to control subjects, whereas ALA administration resulted in a pronounced reduction in ICAM-1 expression in relation to untreated T2DM counterparts (55). AL-Kaisei et al. evaluated the prophylactic effects of ALA (300 mg/kg/day for a duration of 60 days) in a cohort of 30 Sprague–Dawley rats, which were stratified into control, T2DM, and T2DM + ALA groups. The condition of T2DM was elicited through the administration of intraperitoneal streptozotocin injections (40–45 mg/kg) over a period of 3 days. The subjects assigned to the T2DM group exhibited significant elevations in glucose levels, lipid profiles, insulin resistance, and the presence of coronary atherosclerotic plaques. Conversely, the rats that received ALA treatment demonstrated diminished glucose and lipid levels, enhanced insulin sensitivity, and maintained antioxidant enzyme activity (GH-PX), with an absence of coronary atherosclerotic lesions (56). Overall, ALA demonstrates significant cardiovascular and anti-atherosclerotic properties through a multitude of interrelated molecular pathways. ALA mitigates oxidative stress by effectively scavenging ROS and augmenting antioxidant mechanisms, comprising SOD, CAT, GPx, GSH, and the activation of Nrf2. It modulates inflammatory signaling pathways by inhibiting the activation of NF-κB, downregulating pro-inflammatory cytokines such as TNF-α, IL-6, and IL-1β, as well as suppressing adhesion molecules including ICAM-1 and fractalkine in both VSMCs and endothelial cells. Furthermore, ALA enhances lipid metabolism by reducing levels of total cholesterol, LDL, and VLDL, while simultaneously improving HDL ratios. In experimental models of atherosclerosis and myocardial infarction, ALA diminishes apoptosis through the Bax/Bcl-2 and caspase signaling pathways, inhibits macrophage senescence and infiltration, promotes M2b macrophage polarization, and facilitates autophagy, thereby collectively preserving vascular integrity and myocardial functionality. These multifaceted mechanisms underscore ALA’s role as a pleiotropic cardioprotective agent.

### 4.2 ALA and MI

Ischemic cardiomyopathy (ICM) is characterized by oxidative stress, inflammatory processes, and apoptotic mechanisms that contribute to the dysfunction of the left ventricle and the development of myocardial fibrosis. A double-blind, randomized, placebo-controlled study investigated the efficacy of ALA (administered at a dosage of 600 mg/day for a duration of 3 months) as an adjunctive therapeutic intervention in a cohort of 67 diabetic patients diagnosed with ICM. A total of 60 participants successfully completed the trial (ALA *n* = 30; placebo *n* = 30). The administration of ALA resulted in a statistically significant reduction in the levels of inflammatory biomarkers including TNF-α, CRP, TGF-β1, and MMP-2 when compared to the placebo group. Notable improvements were observed in echocardiographic parameters, with a significant increase in left ventricular ejection fraction and a reduction in left ventricular end-systolic diameter, left ventricular end-diastolic diameter, and left atrial diameter (57) (Table 2). The aim of a study was to determine the impacts of ALA (100 mg/kg/day) and mitoquinone (MitoQ, 10 mg/kg/day) preconditioning, both independently and in conjunction, on ischemia–reperfusion injury (IRI) in geriatric Wistar rats (22–24 months). Parameters including myocardial infarct size, oxidative stress biomarkers, mitochondrial functionality, the expression of mitochondrial dynamics-related genes (Mfn1, Mfn2, Foxo1, Drp1, Fis1), hemodynamic variables, and serum lactate dehydrogenase (LDH) levels were rigorously evaluated. The combined administration of ALA and MitoQ notably diminished oxidative stress, reduced LDH concentrations, and lessened infarct size, while concurrently enhancing mitochondrial functionality and reinstating the expression of genes associated with mitochondrial dynamics. The co-administration partially rectified the hemodynamic alterations induced by IRI, thereby illustrating a superior cardioprotective effect in the context of aging hearts compared to the monotherapy interventions (58). Another research endeavored to elucidate the effects of ALA in the context of ischemia/reperfusion injury (I/RI) utilizing H9c2 cardiomyocyte cell lines and rat models subjected to hypoxic/reoxygenation conditions or ligation of the left anterior descending coronary artery. The administration of ALA significantly augmented cellular viability, diminished apoptosis, inhibited the production of ROS and levels of MDA, and enhanced the enzymatic activity of superoxide dismutase. Furthermore, ALA treatment resulted in a reduction of pro-inflammatory cytokines (IL-6, IL-1β, TNF-α) and modulated the expression of apoptotic proteins through a decrease in Bax levels and an increase in Bcl-2 levels. On a mechanistic level, ALA was observed to inhibit the cytoplasmic translocation of high mobility group box 1 (HMGB1), thereby attenuating the activation of the HMGB1/toll-like receptor 4 (TLR4)/NF-κB signaling pathway (59).

**Table 2:** Effects of α-lipoic acid on myocardial ischemia: mechanisms, cellular targets, and animal/human models.

Model/subjects	Intervention	Main findings	Mechanism/pathway	References
60 type 2 diabetic patients with ischemic cardiomyopathy (ICM)	α-Lipoic acid (ALA) 600 mg/day for 3 months (adjunct to standard ICM therapy)	↓ TNF-α, CRP, TGF-β1, MMP-2; ↑ LVEF; ↓ LVESD, LVEDD, LAD	Anti-inflammatory, anti-fibrotic; improved cardiac function	(57)
50 aged male Wistar rats (22–24 months)	ALA 100 mg/kg/day ± MitoQ 10 mg/kg/day for 14 days	↓ oxidative stress, LDH, infarct size; improved mitochondrial function; restored hemodynamics	Enhanced mitochondrial dynamics (↑ Mfn1, Mfn2, Foxo1; ↓ Drp1, Fis1); antioxidant and cardioprotective	(58)
Rat I/R model and H9c2 cells	ALA treatment prior to hypoxia/reoxygenation	↑ cell viability; ↓ apoptosis, ROS, MDA; ↑ SOD; ↓ IL-6, IL-1β, TNF-α; ↓ Bax, ↑ Bcl-2	HMGB1/TLR4/NF-κB signaling; anti-apoptotic, anti-inflammatory, antioxidant	(59)
Type-II diabetic rats (Wistar, 12 weeks, 200–250 g)	ALA 100 mg/kg/day for 5 weeks + ischemic postconditioning (IPostC)	↓ incidence, duration, and severity of ventricular arrhythmias; ↑ connexin-43 expression and NO levels	Anti-arrhythmic; connexin-43 upregulation; nitric oxide-mediated cardioprotection	(60)
Type-II diabetic rats (Wistar, 12 weeks)	ALA 100 mg/kg/day for 5 weeks + IPostC	↓ myocardial infarct size; ↓ LC3, p62 (mRNA and protein); ↓ mitochondrial ROS; restored membrane potential	Modulation of autophagy; improved mitochondrial function; synergistic cardioprotection	(61)
Isolated rat hearts (Langendorff model)	ALA (various doses, 10^−7^ M effective) during ischemia–reperfusion	↓ post-reperfusion arrhythmias; ↑ cardiac sulfane sulfur; improved cardiac function	K(ATP) channel activation; hydrogen sulfide signaling; antioxidant-independent cardioprotection	(62)
H9c2 cardiomyocytes (H/R injury *in vitro*)	ALA pretreatment	↑ cell survival; ↓ total cell death; ↓ autophagy (Beclin-1, LC3II/LC3I)	Autophagy inhibition; cardiomyocyte protection against hypoxia/reoxygenation injury	(63)
Adult male rats (*in vivo* MI/R, 30 min ischemia + 3–72 h reperfusion)	LA pretreatment before coronary ligation	↓ infarct size; ↓ LDH/CK release; ↓ cardiomyocyte apoptosis; preserved cardiac function; ↓ TNF-α and neutrophil accumulation	Activation of PI3K/Akt → Nrf2 nuclear translocation → HO-1 induction; anti-apoptotic and anti-inflammatory	(64)
Isolated rat hearts (Langendorff) + cardiomyocytes (H/R)	LA pretreatment	↑ ALDH2 activity; ↓ apoptosis; ↓ ROS, 4-HNE, MDA; improved cardiac function	ALDH2 activation; PKCε-dependent pathway; antioxidant and anti-apoptotic	(65)
Rat MI/R model (2 h ischemia + reperfusion)	LA 30 min prior to reperfusion	↓ CK activity; ↓ oxidative stress; ↓ apoptosis and inflammation	Multiple target effects: antioxidant, anti-apoptotic, anti-inflammatory; cardioprotection against MI/R injury	(66)
Isolated Langendorff-perfused rat hearts (ischemia 40 min + reperfusion 20–40 min)	Schisandrin B or LA pretreatment (1.2 mmol/kg/day, 3 days)	↓ LDH leakage; ↑ contractile force (Sch B); ↑ myocardial V(E) and V(C)	Modulation of non-enzymatic antioxidants; oxidative stress reduction	(67)
Male Fischer-334 rats (18 mo, *in vivo* I/R: 25 min ischemia + 15 min reperfusion)	Dietary supplementation with VE + alpha-LA (14 wk)	↑ peak arterial pressure during reperfusion; ↓ myocardial lipid peroxidation; improved cardiac performance	ROS scavenging; antioxidant synergy	(68)
Female Sprague–Dawley rats (4 mo, *in vivo* I/R: 25 min coronary occlusion + 10 min reperfusion)	VE + alpha-LA dietary supplementation (14 wk)	↓ lipid peroxidation markers; no significant change in cardiac performance or dysrhythmias	Antioxidant effects reducing oxidative stress during I/R	(69)
Isolated rat hearts and mitochondria (global ischemia + reperfusion)	LA (0.5–10 μM) or DHLA	0.5 μM LA: improved hemodynamics; 10 μM LA: worsened arrhythmias; DHLA ↓ mitochondrial superoxide radicals	Direct ROS scavenging; regulation of mitochondrial O2•− formation; protection of postischemic cardiac function	(70)

ALA, alpha-lipoic acid; ICM, ischemic cardiomyopathy; TNF-α, tumor necrosis factor-alpha; CRP, C-reactive protein; TGF-β1, tissue growth factor-beta1; MMP-2, matrix metalloproteinase-2; LVEF, left ventricular ejection fraction; LVESD, left ventricular end-systolic diameter; LVEDD, left ventricular end-diastolic diameter; LAD, left atrial diameter; MitoQ, mitoquinone; IRI, ischemia/reperfusion injury; IS, infarct size; LDH, lactate dehydrogenase; H/R, hypoxia/reoxygenation; HMGB1, high mobility group box 1,; TLR4, toll-like receptor 4,; NF-κB, nuclear factor-kappa B,; IPostC, ischemic postconditioning; NO, nitric oxide; LC3, microtubule-associated protein 1 light chain 3; ROS, reactive oxygen species; K(ATP), ATP-sensitive potassium channel; PI3K, phosphoinositide 3-kinase; Akt, protein kinase B; Nrf2, nuclear factor erythroid 2-related factor 2; HO-1, heme oxygenase-1; ALDH2, aldehyde dehydrogenase 2; PKCε, protein kinase C epsilon; Sch B, schisandrin B; V(C), ascorbic acid; V(E), alpha-tocopherol; VE, vitamin E; DHLA, dihydrolipoic acid.

Gholami et al. assessed the impact of ALA and ischemic postconditioning (IPostC) on the occurrence of arrhythmias in a model of type-II diabetes mellitus induced in rats. The diabetic condition was established through the administration of a high-fat diet combined with low-dose streptozotocin. Myocardial I/R was performed utilizing the Langendorff apparatus, with ALA (100 mg/kg/day) being provided for a duration of 5 weeks prior to I/R, while IPostC was implemented at the onset of reperfusion. Although IPostC administered in isolation exhibited negligible anti-arrhythmic effects, the synergistic application of ALA preconditioning alongside IPostC markedly diminished the incidence of premature ventricular contractions, ventricular tachycardia, and ventricular fibrillation. This observed cardioprotective effect was correlated with an upregulation of connexin-43 expression and elevated nitric oxide concentrations, suggesting that ALA enhances the therapeutic efficacy of IPostC in diabetic myocardial tissues (60). Mokhtari et al. examined the potential of ALA preconditioning to reinstate the protective effects conferred by IPostC in a model of type-II diabetic rats. The induction of diabetes was achieved through the administration of a high-fat diet in conjunction with low-dose streptozotocin, followed by a regimen of ALA (100 mg/kg/day) for a duration of 5 weeks prior to the ischemia/reperfusion (I/R) procedure. The hearts were subjected to I/R using a Langendorff apparatus, with IPostC being implemented at the onset of reperfusion. The combination of ALA and IPostC resulted in a significant reduction of infarct size, a downregulation of autophagy markers LC3 and p62 at both mRNA and protein levels, and a decrease in mitochondrial ROS as well as membrane depolarization (61). The objective of a research was to elucidate the cardiovascular implications of ALA in isolated rat hearts subjected to IRI utilizing a Langendorff apparatus. ALA was observed to elevate sulfane sulfur concentrations, which play a pivotal role in the release of hydrogen sulfide (H₂S). It is well-established that H₂S mitigates post-reperfusion arrhythmias and affords protection to cardiomyocytes against hypoxia-induced apoptosis, in part by facilitating the activation of ATP-sensitive potassium (K(ATP)) channels. The findings demonstrated that ALA markedly enhanced cardiac function and diminished the incidence of arrhythmias, particularly at a concentration of 10^−7^ M. These cardioprotective effects were completely negated by glibenclamide, a specific inhibitor of K(ATP) channels (62).

Cao et al. examined the impact of ALA on the autophagic process within an *in vitro* hypoxia/reoxygenation (H/R) paradigm of MI/reperfusion injury, utilizing H9c2 cardiomyocyte cell lines. The evaluation of autophagy, a critical mechanism implicated in myocardial I/R damage, was conducted through the quantification of Beclin-1 expression, the ratio of LC3II to LC3I, as well as the assessment of green fluorescent protein-tagged LC3 fusion proteins. The presence of autophagic vacuoles in cells subjected to H/R conditions was substantiated by transmission electron microscopy. The administration of ALA prior to exposure significantly diminished autophagic markers, enhanced cellular viability, and reduced the incidence of overall cell death (63). Deng et al. determined the impacts and underlying mechanisms of ALA in a rat model subjected to *in vivo* MI/R injury. The subjects underwent a duration of 30 min of ischemia, followed by a period of reperfusion, and were administered ALA as a pretreatment. The application of ALA resulted in a significant attenuation of LDH and CK release, a reduction in infarct size, a limitation of cardiomyocyte apoptosis, and an enhancement of cardiac function. Furthermore, it was observed that ALA diminished TNF-α release and neutrophil infiltration within the injured myocardial tissue. Mechanistically, ALA facilitated the phosphorylation of Akt, promoted the nuclear translocation of Nrf2, and augmented the expression of HO-1, while not influencing the activation of p38MAPK or the production of nitric oxide. The inhibition of PI3K was found to negate these protective effects (64). The purpose of a research was to examine whether the cardioprotective properties of ALA are facilitated through the activation of aldehyde dehydrogenase 2 (ALDH2) in the context of IRI. Employing isolated rat hearts and cultured cardiomyocytes, the researchers evaluated cardiac functionality, apoptosis, oxidative stress indicators, and the activity of ALDH2. The conditions of ischemia–reperfusion and hypoxia-reoxygenation resulted in an exacerbation of cardiac dysfunction, increased apoptosis, heightened levels of ROS, and elevated concentrations of toxic aldehydes (specifically, 4-HNE and MDA). Administration of ALA prior to the injury significantly augmented ALDH2 activity, ameliorated cardiac function, and mitigated oxidative stress and apoptosis. These protective effects were negated in the presence of inhibitors targeting ALDH2 or PKCε (65).

Wang et al. analyzed the protective effects and underlying mechanisms of ALA in the context of MI/R injury in a rat model. MI/R notably elevated the activity of creatine kinase (CK), intensified oxidative stress, diminished the activity of antioxidant enzymes, and facilitated apoptosis and inflammation in a time-dependent manner. The administration of ALA 30 min prior to reperfusion significantly mitigated these alterations. ALA diminished the production of ROS, reinstated the antioxidant defense mechanisms, and inhibited apoptotic and inflammatory responses. These results indicated that ALA confers protection against MI/R-induced cardiac injury via antioxidant, anti-apoptotic, and anti-inflammatory pathways. The study enhances our comprehension of ALA’s pharmacological properties and endorses its prospective therapeutic application in I/RI and cardiovascular diseases (66). Ko et al. employed isolated Langendorff-perfused rat hearts to scrutinize IR injury and the contributions of non-enzymatic antioxidants. A 40-min ischemic interval succeeded by reperfusion resulted in considerable myocardial injury, evidenced by elevated LDH efflux, diminished contractile strength, and lowered ascorbic acid (vitamin C) concentrations. The levels of reduced glutathione and alpha-tocopherol (vitamin E) exhibited stability except under exacerbated IR circumstances. Administration of schisandrin B (Sch B) or ALA as a pretreatment for 3 days conferred protection against IR injury. Sch B demonstrated superior enhancement of contractile recovery, whereas ALA was more proficient in attenuating LDH efflux. Both interventions elevated myocardial concentrations of vitamin C and E, indicating that cardioprotective mechanisms are, at least in part, associated with the modulation of non-enzymatic antioxidant defenses during episodes of oxidative stress (67). Coombes et al. explored the impacts of dietary antioxidant supplementation utilizing vitamin E (VE) and ALA on myocardial ischemia–reperfusion (I-R) responses in senescent male Fischer-334 rats. The subjects were administered either a control diet or a VE/ALA fortified diet for a duration of 14 weeks prior to being subjected to 25 min of ischemia, succeeded by 15 min of reperfusion. The ANTIOX-supplemented rats demonstrated markedly elevated peak arterial pressure during the reperfusion phase and exhibited protection against I-R-induced myocardial lipid peroxidation. Furthermore, heart homogenates derived from ANTIOX rats displayed a reduction in oxidative damage *in vitro* (68).

Coombes et al. evaluated the impacts of vitamin E (VE) and ALA supplementation on cardiac functionality, dysrhythmias, and biochemical alterations during *in vivo* ischemia–reperfusion (I-R) in 4-month-old female Sprague–Dawley rats. The subjects were administered either a control diet or a VE/ALA-enriched diet over a duration of 14 weeks. Notwithstanding the considerable elevation of myocardial VE concentrations in the supplemented cohort, no discernible differences were detected in cardiac performance or the frequency of ventricular dysrhythmias during I-R. Nevertheless, indicators of lipid peroxidation were significantly diminished in the supplemented rats, suggesting a protective role of antioxidants against oxidative stress without influencing cardiac functionality (69). Another study shown the ramifications of ALA and its reduced derivative, dihydrolipoic acid (DHLA), on cardiac IRI at both the organ and mitochondrial tiers. Isolated rat cardiac tissues were exposed to global ischemia followed by reperfusion with varying concentrations of ALA. Administration of low-dose ALA (0.5 μM) facilitated hemodynamic recovery, whereas high-dose ALA (10 μM) exacerbated recovery and extended rhythm disturbances. DHLA administration to isolated mitochondria did not mitigate respiratory dysfunction but significantly diminished mitochondrial superoxide (O2•−) generation. Furthermore, DHLA indirectly modulated O2•− production through the redox-cycling mechanism of ubiquinone (70). Overall, ALA demonstrates cardioprotective properties in the context of myocardial I/R injury through a diverse array of molecular mechanisms. ALA alleviates oxidative stress by effectively scavenging ROS, augmenting the activity of antioxidant enzymes, and modulating non-enzymatic antioxidants, including vitamins C and E. It attenuates apoptosis by manipulating the ratios of Bax to Bcl-2, constraining mitochondrial membrane depolarization, and activating critical signaling pathways such as ALDH2, PI3K/Akt, and Nrf2/HO-1. ALA diminishes inflammation through the inhibition of TNF-α, IL-6, IL-1β, and the HMGB1/TLR4/NF-κB signaling cascade. It enhances mitochondrial functionality by regulating genes pertinent to mitochondrial dynamics (Mfn1/2, Drp1, Fis1) and decreasing mitochondrial ROS. Furthermore, ALA influences autophagy (LC3, p62, Beclin-1), promotes H₂S release via K(ATP) channels, and works in concert with ischemic postconditioning and antioxidants such as vitamin E or MitoQ, collectively improving cardiac performance, decreasing infarct size, and averting arrhythmias during I/R injury.

### 4.3 ALA and myocardial infarction

MI is linked to significant morbidity and mortality, while the macrophage senescence-associated secretory phenotype (SASP) is integral to the healing process following infarction. An investigation explored the correlation between ALA and macrophage senescence through the application of single-cell RNA sequencing (GSE163465), molecular assessments, coculture methodologies, and experimental animal models. The findings demonstrated an age-independent augmentation in senescent macrophages during MI, marked by increased levels of H2A X, CCL7, IL-1β, and CDKN1A, alongside diminished SOD2, signifying oxidative stress and mitochondrial impairment. ALA administration inhibited the degradation of SIRT1, facilitated the nuclear translocation of Nrf2, decreased the production of ROS and autophagy flux, and mitigated the expression of SASP (71) (Table 3). The aim of a study was to determine the impact of concomitant supplementation with ALA and mitoquinone (MitoQ) on cardiac functionality in aged rats following MI. The experimental rats were subjected to a 30-min occlusion of the left anterior descending artery, followed by a subsequent 24-h reperfusion period, subsequent to receiving ALA and MitoQ for a duration of 2 weeks prior to the induction of ischemia. The dual intervention resulted in a statistically significant enhancement of cardiac function in comparison to control subjects. These observed advantages were correlated with notable declines in pro-inflammatory cytokines (TNF-α, IL-6, IL-1β) and markers of apoptosis (Bax, caspase-3, cytochrome c), in addition to a reduced incidence of TUNEL-positive cells (72). Gholami et al. evaluated the synergistic impacts of ALA and ischemic postconditioning (Post) on myocardial infarction and apoptosis in rats suffering from chronic type II diabetes that were subjected to I/R injury. The diabetic condition was established through a high-fat diet and administration of streptozotocin, with rats receiving ALA for a duration of 5 weeks prior to the I/R event. Although Post alone did not exhibit a statistically significant reduction in infarct size or levels of LDH, ALA administered alone demonstrated protective properties. Importantly, the combined administration of ALA and Post treatment significantly reduced apoptotic markers, such as Bax, the Bax/Bcl2 ratio, and cleaved caspase-3, while simultaneously enhancing Bcl2 expression. Additionally, histopathological assessments showed marked improvements (73).

**Table 3:** Effects and mechanisms of α-lipoic acid in myocardial infarction: preclinical and clinical studies.

Model/subjects	Intervention	Key findings/mechanisms	Outcome	References
Mouse MI model; macrophages under hypoxia	ALA treatment; scRNA-seq, qPCR, Western blot, immunofluorescence	↑ H2A. X, ↑ CCL7, ↑ IL1β, ↑ CDKN1A, ↓ SOD2, ↑ ROS, ↓ SIRT1 degradation (via ALA), ↑ Nrf2 nuclear translocation, ↓ autophagy flux	Alleviated macrophage senescence and myocardial ischemic injury; improved cardiac repair	(71)
Aged rats; MI induced by LADA occlusion/reperfusion	ALA (100 mg/kg, oral) + Mito Q (10 mg/kg, IP) for 2 weeks pre-ischemia	↓ TNF-α, ↓ IL-6, ↓ IL-1β, ↓ Bax, ↓ caspase-3, ↓ Cyt-c, ↓ TUNEL-positive cells	Synergistic reduction of cardiac dysfunction via suppression of inflammation and apoptosis in aged MI rats	(72)
Diabetic rats (high-fat diet + streptozotocin); I/R injury	ALA (100 mg/kg/day, oral, 5 weeks) ± ischemic postconditioning (6 × 10/10 s cycles)	↓ Bax, ↓ Bax/Bcl2, ↓ cleaved caspase-3, ↑ Bcl2	Inhibited apoptosis and promoted cardiac recovery after I/R in diabetic hearts	(73)
MI mice; macrophages under hypoxia; M1 polarization induced by LPS + IFN-γ	ALA treatment; analysis of cardiomyocyte function, cytokines, apoptosis, autophagy, ROS, MMP	↑ M2b polarization, ↓ inflammatory cytokines, ↓ ROS, ↓ MMP, ↓ apoptosis, ↓ autophagy, ↓ HMGB1/NF-κB pathway	Alleviated MI and improved cardiac recovery via M2b macrophage polarization and HMGB1/NF-κB modulation	(74)
Acute MI mice; primary cardiomyocytes under H₂O₂	LA@PLGA patch films via electrospinning for controlled LA release	↓ ROS, ↓ oxidative stress, ↓ apoptosis, ↓ senescence, ↓ DNA damage, ↓ cytokine-mediated processes, ↓ ferroptosis	Protected heart from MI-induced damage, improved function, reduced fibrosis	(75)
112 patients with type 2 diabetes and history of non-Q MI	Oral ALA for 4 months alongside standard therapy (ACE inhibitor, β-blocker, statin, antiplatelet)	↓ CRP, ↓ IL-6, ↓ TNF-α, ↔ IL-10	Reduced systemic inflammation in type 2 diabetes patients post-MI, supporting cardiovascular protection	(76)
Rats: control, ISO, LA + ISO, STZ + ISO, STZ + LA + ISO	LA (10 mg/kg/day) for 14 days	In non-diabetic MI rats: ↓ cardiac necrosis, ↓ leucocyte infiltration, ↓ lipid peroxidation, ↑ paraoxonase and lactonase activities. In diabetic MI rats: ↔ histopathologic and biochemical parameters	Prevented MI in non-diabetic rats; insufficient effect in diabetic rats	(77)
Mice exposed to intermittent hypoxia; H9C2 cardiomyocytes	LA treatment *in vivo* and *in vitro*	↑ Nrf2 nuclear translocation, ↑ autophagy, ↓ apoptosis, ↑ cell viability	Alleviated IH-induced myocardial injury; improved cardiac function via Nrf2-mediated autophagy	(78)
C57Bl/6 mice; AMI induced by LCA occlusion	Daily ALA (15 or 75 mg/kg/day) pre- and post-AMI	↓ infarct size, ↑ LVEF, ↓ LVESV, ↑ survival rate	Protected against AMI, attenuated left ventricular remodeling, preserved cardiac function	(79)

ALA, α-lipoic acid; MI, myocardial infarction; scRNA-seq, single-cell RNA sequencing; qPCR, quantitative polymerase chain reaction; SASP, senescence-associated secretory phenotype; SOD2, superoxide dismutase 2; ROS, reactive oxygen species; MMP, mitochondrial membrane potential; I/R, ischemia/reperfusion; LADA, left anterior descending artery; Bax, Bcl-2-associated X protein; Bcl2, B-cell lymphoma 2; Cyt-c, cytochrome c; TUNEL, terminal deoxynucleotidyl transferase dUTP nick end labeling; HMGB1, high mobility group box 1; NF-κB, nuclear factor kappa B; PLGA, poly(lactic-co-glycolic) acid; LA, lipoic acid; CRP, C-reactive protein; IL, interleukin; TNF-α, tumor necrosis factor-alpha; ISO, isoproterenol; STZ, streptozotocin; H9C2, rat cardiomyoblast cell line; IH, intermittent hypoxia; OSAS, obstructive sleep apnea syndrome; AMI, acute myocardial infarction; LVR, left ventricular remodeling; LVESV, left ventricular end-systolic volume; LVEF, left ventricular ejection fraction.

Wang et al. examined the implications of ALA in the context of MI, with particular emphasis on the modulation of macrophage activity and the subsequent cardiac recuperation. Myocardial infarction was experimentally induced in murine models via ligation of the left anterior descending artery, and macrophages were exposed to conditions of hypoxia alongside stimuli promoting M1 polarization. Treatment with ALA facilitated the transition toward M2b macrophage polarization while concurrently attenuating the production of pro-inflammatory cytokines under hypoxic circumstances. Additionally, ALA treatment resulted in a diminution of ROS generation and the preservation of mitochondrial membrane potential *in vitro*. The supernatants derived from ALA-treated macrophages exhibited a capacity to diminish apoptosis and autophagy in hypoxic cardiomyocytes. On a mechanistic level, ALA effectively inhibited the activation of the HMGB1/NF-κB signaling cascade (74). Xie et al. demonstrated an innovative drug delivery mechanism to augment the therapeutic potency of ALA in the context of acute myocardial infarction (AMI). In light of LA’s swift elimination and extensive biodistribution, the researchers meticulously crafted a controlled-release thin film (ALA@PLGA) employing poly(lactic-co-glycolic acid) through the process of electrospinning. This advanced system facilitated prolonged localized release of ALA, thereby proficiently neutralizing ROS within the compromised myocardial tissue. *In vitro* experiments demonstrated that ALA@PLGA significantly diminished oxidative stress and apoptosis in cardiomyocytes subjected to hydrogen peroxide treatment. In murine models of AMI, the epicardial application of this film markedly enhanced cardiac functionality and mitigated fibrosis throughout the ventricular remodeling phase. Furthermore, it effectively reduced oxidative stress, cellular senescence, DNA damage, inflammatory responses, apoptosis, and ferroptosis (75). The aim of a study was to explore the ramifications of ALA in individuals diagnosed with T2DM, particularly those possessing a prior history of non-Q myocardial infarction, a demographic characterized by elevated risks of recurrent myocardial infarction and cardiovascular mortality. A cohort comprising 112 patients undergoing conventional therapeutic regimens which included ACE inhibitors, β-blockers, statins, antiplatelet agents, and oral hypoglycemic medications, was assessed. Following a four-month regimen of ALA supplementation, participants exhibited notable decreases in systemic inflammatory markers, such as C-reactive protein, IL-6, and TNF-α, whereas the concentrations of the anti-inflammatory cytokine IL-10 exhibited no significant alterations (76).

Ozgun et al. examined the cardioprotective properties of LA in the context of isoproterenol-induced myocardial infarction in both diabetic and non-diabetic rat models. The induction of diabetes was accomplished via the administration of streptozotocin, followed by a regimen of LA (10 mg/kg/day) for a duration of 14 days prior to the induction of myocardial injury. The administration of isoproterenol resulted in heightened cardiac necrosis, leukocyte infiltration, and serum lipid peroxidation, concomitantly leading to a reduction in the activities of paraoxonase and lactonase. In non-diabetic rats afflicted with myocardial infarction, LA markedly attenuated cardiac necrosis, inflammation, and lipid peroxidation, while also reinstating the activities of antioxidant enzymes. Conversely, in diabetic rats subjected to myocardial infarction, LA did not demonstrate a statistically significant improvement in either histopathological or biochemical parameters (77). Another study determined the therapeutic efficacy of ALA in the context of myocardial damage linked to obstructive sleep apnea syndrome, which is characterized by intermittent hypoxia (IH). *In vivo* studies demonstrated that exposure to IH compromised cardiac function in murine models, whereas administration of ALA significantly ameliorated cardiac performance. Mechanistically, ALA was found to activate the Nrf2 signaling pathway and facilitate autophagy. *In vitro* experiments revealed that IH induced apoptotic processes and decreased viability in H9C2 cardiomyocytes, while ALA enhanced Nrf2 nuclear translocation, activated downstream antioxidant signaling pathways, augmented autophagy, and inhibited apoptotic events. The suppression of Nrf2 through the application of ML385 resulted in a reduction of autophagy and attenuated the cardioprotective effects of ALA, thereby corroborating the role of Nrf2 in this context (78). Another study assessed the efficacy of daily administration of ALA in providing protection against AMI and the ensuing left ventricular remodeling (LVR). In murine subjects that were pre-treated with ALA for a duration of 7 days prior to the induction of coronary artery occlusion, a significant reduction in infarct size was observed in comparison to the control group. In a distinct study focusing on LVR, the initiation of ALA treatment 1 day post-AMI and its continuation over an extended period resulted in notable improvements in left ventricular end-systolic volume and ejection fraction at both 28 and 56 days following the infarction event. Moreover, survival rates demonstrated a marked enhancement in the cohort of mice that received ALA treatment (79). Collectively, the aggregated findings from these studies elucidate that ALA confers cardioprotective properties via a complex interplay of antioxidant, anti-inflammatory, anti-apoptotic, and mitochondrial-regulatory mechanisms. ALA diminishes the production of ROS, facilitates Nrf2 nuclear translocation, and enhances the expression of downstream antioxidant defenses, thus mitigating damage induced by oxidative stress. It attenuates inflammatory signaling pathways by inhibiting the HMGB1/NF-κB cascade and reducing the levels of pro-inflammatory cytokines such as TNF-α, IL-6, and IL-1β. ALA mitigates apoptosis through the modulation of the Bax/Bcl-2 ratio, decreasing the activation of caspase-3, and maintaining the integrity of mitochondrial membrane potential. Additionally, it fosters advantageous M2b, inhibits the SASP, and regulates the dynamics of autophagic flux. In conjunction with other therapeutic strategies (e.g., in combination with MitoQ or ischemic postconditioning), ALA synergistically enhances cardiac function and diminishes infarct size. Prolonged delivery systems further augment its therapeutic efficacy in the context of myocardial infarction and associated cardiac injuries.

Cellular studies consistently demonstrate ALA’s ability to attenuate oxidative injury across diverse cardiovascular-relevant cell types, including cardiomyocytes, endothelial cells, and smooth muscle cells. Unlike compounds that rely solely on direct antioxidant scavenging, ALA enhances intracellular antioxidant systems by regenerating glutathione, vitamin C, and vitamin E. This is accompanied by activation of Nrf2-dependent transcriptional programs, as shown by reporter assays and gene-expression profiling. Methodologically, mitochondrial membrane potential assays, Seahorse respirometry, and ROS quantification techniques repeatedly show improved mitochondrial integrity and reduced oxidative burden after ALA treatment. These findings position ALA as a broader metabolic modulator rather than a simple antioxidant. Animal studies extend these findings by demonstrating functional improvements in cardiac performance and vascular physiology. In rodent models of ischemia–reperfusion injury, diabetic cardiomyopathy, and hypertension, ALA supplementation reduces myocardial infarct size, improves endothelial relaxation, and enhances mitochondrial enzyme activity—particularly within pyruvate dehydrogenase and alpha-ketoglutarate dehydrogenase complexes. Biochemical analyses consistently report decreases in lipid peroxidation markers (e.g., MDA) and increases in endogenous antioxidant enzyme activities. Notably, ALA’s redox-cycling capacity distinguishes it from non-redox-active antioxidants, enabling sustained protection under high oxidative load.

Human studies, although more variable, generally support the cardiometabolic benefits observed in preclinical models. Clinical trials in diabetic and metabolic-syndrome populations show improvements in endothelial function, reductions in oxidative stress biomarkers, and enhanced insulin sensitivity—an outcome relevant to cardiovascular risk. Variability in dosing regimens and oral bioavailability, however, introduces heterogeneity in effect size. Nonetheless, the consistency of redox-related improvements across trials suggests a robust underlying mechanism. Collectively, evidence across experimental systems supports ALA as a multifaceted modulator of mitochondrial redox biology in cardiovascular disease. By combining direct antioxidant actions, regeneration of endogenous defense systems, metabolic cofactor activity, and activation of cytoprotective signaling pathways, ALA occupies a unique therapeutic niche among mitochondrial-targeted interventions. Future comparative trials and dose-standardized clinical studies will be critical to defining its optimal application in cardiovascular prevention and therapy.

## 5 UA as a mitophagy-inducing nutritional therapeutic

An investigation examined the cardioprotective properties of UA, a derivative of ellagitannins produced by gut microbiota, in the context of MIR injury, a significant factor in the pathogenesis of AMI. The outcomes of both *in vitro* and *in vivo* analyses indicated that UA enhanced cardiac performance, diminished oxidative stress, alleviated mitochondrial impairment, and suppressed ferroptosis during MIR. At the mechanistic level, UA was found to elevate the expression of Nrf2, facilitate its translocation to the nucleus, and activate subsequent antioxidant defense mechanisms. The obstruction of Nrf2 function negated the protective effects of UA, thereby underscoring its pivotal role in this context (80) (Table 4). The aim of a study was to determine the implications of UA, a polyphenolic metabolite originating from the gut, on the transformation of cardiac fibroblasts to myofibroblasts (CMT) in the context of myocardial fibrosis subsequent to MI. *In vitro* analyses demonstrated that UA significantly inhibited fibroblast proliferation, migration, and invasion induced by TGF-β1. In terms of underlying mechanisms, UA was found to activate the Nrf2 signaling pathway, while the knockdown of Nrf2 partially mitigated these protective outcomes. *In vivo* experiments revealed that the administration of UA in a rat model of MI resulted in the upregulation of Nrf2, a decrease in myocardial damage, and a reduction in the extent of fibrosis (81). Tang et al. explored the cardioprotective properties of UA in the context of myocardial I/R injury through both *in vivo* and *in vitro* methodologies. In murine models subjected to I/R, as well as in cardiomyocytes subjected to hypoxia/reoxygenation, UA was observed to significantly diminish infarct size, reduce cellular mortality, and attenuate myocardial apoptosis. Furthermore, UA was found to enhance the antioxidant capacity within cardiomyocytes, thereby alleviating oxidative stress provoked by I/R episodes. From a mechanistic perspective, the cardioprotective effects of UA were revealed to be contingent upon the PI3K/Akt signaling pathway, with the use of LY294002 effectively negating its protective benefits (82).

**Table 4:** Summary of mechanistic and functional effects of urolithin A across cardiovascular, metabolic, and endothelial models.

Experimental model	Molecular targets/pathway	Effects on oxidative stress, ferroptosis, fibrosis, and metabolism	Effects on cardiac function/injury	References
*In vitro* and *in vivo* ischemia/reperfusion models	Nrf2 signaling pathway	↓ oxidative stress; ↓ mitochondrial damage; ↓ ferroptosis; ↑ Nrf2 nuclear translocation and downstream antioxidant defenses	Enhanced cardiac function; reduced myocardial ischemia–reperfusion injury	(80)
TGF-β1-treated primary rat cardiac fibroblasts; rat MI model (LAD ligation)	Nrf2 pathway	↓ cardiac fibroblast-to-myofibroblast transformation (CMT); ↓ cell proliferation, migration, invasion; ↑ Nrf2 expression	Reduced myocardial fibrosis; improved histological markers of myocardial damage	(81)
*In vitro* hypoxia/reoxygenation; *in vivo* mouse myocardial I/R	PI3K/Akt signaling pathway	↑ antioxidant capacity; ↓ myocardial apoptosis	↓ infarct size; ↓ cell death; improved myocardial recovery; effects blocked by PI3K/Akt inhibitor LY294002	(82)
Mouse SACI model; *in vitro* cardiomyocytes	Mitochondrial fatty acid oxidation; CPT1; mitochondrial membrane potential	↓ oxidative stress; ↓ myocardial apoptosis; restored mitochondrial metabolism; ↑ ATP production	Attenuated cardiac mitochondrial dysfunction; reduced myocardial cell death and tissue damage	(83)
Streptozotocin-induced diabetic rats	Pro-inflammatory cytokine fractalkine; calcium handling proteins (SERCA, phospholamban)	↓ fractalkine expression; improved cardiomyocyte microenvironment; recovery of calcium dynamics	↑ ventricular pressure rise; ↓ isovolumic contraction time; improved cardiomyocyte contractility and re-lengthening; UB slightly more effective due to myocardial accumulation	(84)
Mouse renal ischemia–reperfusion model; *in vitro* hypoxia cells	p62-Keap1-Nrf2 signaling pathway	↓ ROS; ↑ nuclear Nrf2; promoted autophagy; ↓ tubular cell apoptosis	Protection against acute kidney injury; improved renal tissue integrity	(85)
ApoE^−^/^−^ mice fed high-fat/high-cholesterol diet; TNF-α-stimulated HUVECs	NO production; YAP/TAZ; TEAD transcription; SREBP1/2 in liver	↓ macrophage content and endothelial activation; ↓ intraplaque hemorrhage and necrotic core; ↑ smooth muscle actin; improved cholesterol metabolism	Increased plaque stability; reduced atherosclerotic lesion size; improved vascular integrity	(86)
Wistar rats fed high-cholesterol diet + Vitamin D₃; balloon aortic injury	SR-BI; Nrf2; p-ERK1/2	↓ plasma lipid and Ang II levels; ↑ SR-BI expression; ↓ p-ERK1/2	Reduced aortic lesion formation; improved vascular structure	(87)
Human artery endothelial cells incubated with ox-LDL	miR-27; ERK/PPAR-γ pathway; eNOS/NO	↑ NO and eNOS; ↓ ICAM-1, MCP-1, TNF-α, IL-6, endothelin-1; ↑ PPAR-γ; ↓ miR-27; ↓ p-ERK1/2	Attenuated monocyte adhesion; improved endothelial function under oxidative stress	(88)

UA, Urolithin A; UB, Urolithin B; I/R, ischemia/reperfusion; MIR, myocardial ischemia–reperfusion; MI, myocardial infarction; SACI, severe acute pancreatitis-associated cardiac injury; CPT1, carnitine palmitoyltransferase 1; ROS, reactive oxygen species; SERCA, sarcoplasmic reticulum calcium ATPase; HCAEC, human coronary artery endothelial cells; AKI, acute kidney injury; RIR, renal ischemia–reperfusion; ApoE^−^/^−^, Apolipoprotein E-deficient; HUVEC, human umbilical vein endothelial cells; NO, nitric oxide; YAP/TAZ, yes-associated protein/transcriptional coactivator with PDZ-binding motif; TEAD, TEA domain transcription factor; SREBP1/2, sterol regulatory element-binding protein 1/2; SR-BI, scavenger receptor class B type I; Ang II, angiotensin II; ox-LDL, oxidized low-density lipoprotein; ICAM-1, intercellular adhesion molecule 1; MCP-1, monocyte chemoattractant protein 1; PPAR-γ, peroxisome proliferator-activated receptor gamma; ERK, extracellular signal-regulated kinase; p-ERK1/2, phosphorylated ERK1/2.

Yang et al. evaluated the protective attributes of UA in the context of severe acute pancreatitis–associated acute cardiac injury (SACI). Utilizing a mouse model of SACI and accompanying *in vitro* analyses, findings indicated that SACI induced mitochondrial dysfunction, metabolic dysregulation, oxidative stress, and apoptosis of cardiomyocytes. Administration of UA led to a notable reduction in both pancreatic and cardiac tissue injury, a decrease in the levels of lipase, amylase, and pro-inflammatory cytokines, as well as a mitigation of myocardial apoptosis and oxidative stress. From a mechanistic standpoint, UA was found to restore mitochondrial functionality through the enhancement of mitochondrial membrane potential, ATP synthesis, and the normalization of fatty acid oxidation processes. The inhibition of CPT1 was observed to compromise these protective effects, thereby implicating CPT1 as a crucial mediator in this context (83). The aim of a research was to assess the impact of UA and UB, metabolites derived from ellagitannins present in the gut, on the onset of early diabetic cardiomyopathy in rats with type 1 diabetes induced by streptozotocin. The administration of urolithins resulted in a reduction of myocardial expression of the pro-inflammatory cytokine fractalkine by approximately 30%, thereby mitigating inflammation associated with hyperglycemia. Functionally, both UA and UB demonstrated enhancements in cardiac performance, evidenced by an increase in ventricular pressure rise, a reduction in isovolumic contraction time, and an augmentation in cardiomyocyte contractility. Notable advancements in calcium handling were observed, including accelerated re-lengthening rates, abbreviated re-lengthening durations, and enhanced efficiency in cytosolic calcium clearance. Urolithin B exhibited marginally superior outcomes, presumably attributable to a greater myocardial accumulation of UB and its associated metabolites (84).

Zhang et al. examined the neuroprotective properties of UA in the context of renal ischemia–reperfusion (RIR)–induced acute kidney injury (AKI) in murine models. *In vitro* analyses revealed that UA administration resulted in a reduction of p62 and Keap1 levels, concomitantly elevating nuclear Nrf2, thereby signifying the activation of the p62-Keap1-Nrf2 signaling cascade. *In vivo* experiments demonstrated that UA mitigated tubular cell apoptosis, diminished the levels of ROS, and augmented autophagic processes within the renal tissues post-RIR. From a mechanistic perspective, UA effectively mitigates oxidative stress and facilitates autophagic degradation via the p62-Keap1-Nrf2 pathway, thereby conferring protection to renal tissue from the deleterious effects of IRI (85). Another investigation examined the influence of UA, a metabolite derived from gut microbial processing of ellagitannins, on the progression of atherosclerosis and the stability of atherosclerotic plaques in ApoE-deficient murine models. Administration of UA resulted in a significant reduction in atherosclerotic lesions, macrophage accumulation, size of the necrotic core, incidence of intraplaque hemorrhage, and expression levels of endothelial adhesion molecules, concomitantly enhancing smooth muscle actin levels and thickness of the fibrous cap, thus indicating an improvement in plaque stability. At the mechanistic level, UA effectively inhibited TNF-α-induced endothelial cell activation and monocyte adhesion in human umbilical vein endothelial cells (HUVECs) by facilitating the production of nitric oxide and suppressing YAP/TAZ-TEAD signaling pathways, independent of NF-κB activation. Furthermore, UA exerted regulatory effects on hepatic SREBP1/2 transcription and processing, thereby enhancing cholesterol metabolic pathways (86).

A research determined the anti-atherosclerotic properties of UA in Wistar rats that were subjected to a diet high in cholesterol and aortic balloon injury. The administration of UA (3 mg/kg/day) over a duration of 12 weeks resulted in a significant diminution of plasma lipid and angiotensin II (Ang II) concentrations, as well as an amelioration of aortic lesions. At a mechanistic level, UA was found to upregulate the expression of scavenger receptor class B type I (SR-BI) while concurrently inhibiting the phosphorylated ERK1/2 (p-ERK1/2) signaling pathway. Notably, the expression of SR-BI demonstrated an inverse correlation with Ang II levels, implying that UA facilitates cholesterol clearance and vascular protection via the induction of SR-BI and the suppression of the ERK1/2 signaling pathway (87). Another work examined the protective properties of UA in the context of ox-LDL–induced endothelial dysfunction. UA exhibited a dose-dependent enhancement of nitric oxide synthesis and endothelial nitric oxide synthase (eNOS) activity, concurrently diminishing the expression of adhesion molecules (ICAM-1, MCP-1) and inhibiting monocyte adhesion. Furthermore, it attenuated the levels of pro-inflammatory cytokines (TNF-α, IL-6, endothelin-1) while concurrently upregulating the mRNA expression of PPAR-γ. At the mechanistic level, UA was found to decrease the expression of miR-27, and the overexpression of miR-27 was observed to negate the effects of UA on PPAR-γ, thereby identifying miR-27 as a significant mediator. Moreover, UA was shown to inhibit phosphorylated ERK1/2, which contributed to the reduction of IL-6 levels and the enhancement of PPAR-γ expression (88). These investigations collectively elucidate that UA confers protection to cardiovascular and renal systems through a multitude of interrelated molecular pathways. UA activates the Nrf2 signaling cascade, thereby augmenting antioxidant defenses, diminishing ROS, and facilitating autophagy, which collectively alleviates IRI and acute renal impairment. It mitigates mitochondrial dysfunction and apoptosis by maintaining mitochondrial membrane potential and ATP synthesis, as well as modulating the PI3K/Akt signaling pathway, which results in a reduction of cell death within cardiomyocytes. Furthermore, UA manifests anti-inflammatory properties through the inhibition of TNF-α, IL-6, IL-1β, ICAM-1, and MCP-1, while also regulating miR-27/PPAR-γ and YAP/TAZ-TEAD signaling pathways, thereby enhancing endothelial functionality. In the context of atherosclerosis, UA contributes to plaque stabilization through the upregulation of SR-BI and markers indicative of fibrous caps, concurrently downregulating ERK1/2 and lipogenic transcription factors such as SREBP1/2. In summary, UA serves a protective role in tissues via mechanisms that are antioxidant, anti-apoptotic, anti-inflammatory, mitochondrial, and lipid-regulating in nature.

Cellular investigations have provided strong mechanistic evidence supporting UA-induced mitophagy. In skeletal muscle cells, cardiomyocytes, and neuronal models, UA treatment consistently activates key mitophagy regulators, including the PINK1/Parkin signaling pathway. Experimental approaches such as fluorescence-based mitophagy reporters, confocal microscopy, and Western blot analysis of autophagy markers demonstrate increased mitochondrial turnover following UA exposure. These studies often report improved mitochondrial membrane potential, enhanced respiratory capacity, and reduced accumulation of damaged mitochondrial components. Compared with classical antioxidants such as ALA, UA shows relatively modest direct ROS-scavenging activity but produces pronounced effects on mitochondrial network remodeling and quality control. Animal studies further support the physiological relevance of these mechanisms. In rodent and invertebrate models of aging, metabolic dysfunction, and muscle degeneration, UA supplementation stimulates mitophagy and improves mitochondrial function. Methodologically, these studies combine molecular analyses of mitophagy markers with functional assessments such as endurance capacity, muscle strength measurements, and mitochondrial respiration assays. UA administration has been associated with increased expression of mitochondrial biogenesis regulators such as PGC-1α and NRF1, suggesting coordinated activation of mitochondrial renewal pathways. Importantly, improvements in muscle performance and metabolic parameters have been reported in several models, highlighting the functional consequences of enhanced mitochondrial turnover. Translational studies in humans are relatively recent but provide encouraging evidence. Clinical trials evaluating UA supplementation have demonstrated activation of mitochondrial gene expression programs in skeletal muscle, along with increases in biomarkers associated with mitochondrial biogenesis and cellular energy metabolism. In some studies, improvements in muscle endurance and mitochondrial efficiency have also been observed. These trials typically employ transcriptomic analyses, metabolomic profiling, and functional performance testing, providing mechanistic and physiological endpoints that support UA’s role as a mitochondrial health modulator.

Overall, the body of evidence suggests that UA represents a distinct class of nutritional therapeutics targeting mitochondrial quality control rather than direct antioxidant defense. By stimulating mitophagy and promoting mitochondrial renewal, UA addresses a fundamental aspect of mitochondrial dysfunction that contributes to aging and cardiometabolic disease. While the mechanistic consistency across experimental models is compelling, further long-term clinical studies are required to determine the durability of UA-induced mitochondrial improvements and their direct impact on cardiovascular outcomes.

## 6 EGT: an emerging cytoprotective antioxidant in cardiometabolic disorders

An investigation assessed the hypothesis that EGT supplementation may enhance endothelial dysfunction, mitigate macrophage inflammatory activity under hyperlipidemic conditions, and decrease atherosclerotic risk markers in mice subjected to a diet-induced obesity model. The administration of EGT did not influence endothelial tube formation or structural integrity; however, it did result in a reduction of nitric oxide concentrations within endothelial cells. Furthermore, it diminished the accumulation of ROS and potentially modulated nitric oxide synthesis in macrophages. Nevertheless, in the context of obese mice, EGT demonstrated no significant alterations in circulating lipid levels, lipoproteins, or glucose concentrations. Collectively, EGT exhibited moderate vascular effects *in vitro*, yet conferred limited systemic cardiometabolic advantages *in vivo* (89) (Table 5). MI represents a predominant contributor to cardiovascular-related mortality, while soluble fms-like tyrosine kinase-1 (sFlt-1) plays a significant role in post-MI cardiac injury. Another research examined the cardioprotective mechanisms exerted by EGT within an experimental rat model of MI. Administration of 10 mg/kg EGT for a duration of 7 days yielded enhancements in cardiac function, diminished infarct size, restricted adverse remodeling and fibrosis, and mitigated cardiomyocyte apoptosis. EGT markedly decreased cardiac levels of MCP-1, p65, and phosphorylated p65, alongside a reduction in circulating sFlt-1 concentrations. Immunofluorescence analyses revealed that EGT effectively mitigated MI-induced GLRX expression within peri-coronary regions. In hypoxic human coronary artery endothelial cells, EGT led to a reduction in the expression levels of sFlt-1, GLRX, and Wnt5a (90). The aim of a study was to assess the ramifications in isolated Langendorff-perfused rabbit hearts that were exposed to a duration of 45 min of global ischemia, subsequently followed by a 30-min reperfusion period. EGT (10^−5^ M and 10^−4^ M), administered prior to the onset of ischemia and during the reperfusion phase, did not confer significant cardioprotective effects. It did not enhance the recovery of developed pressure, mitigate diastolic dysfunction, nor diminish the release of creatine kinase and lactate. Moreover, it failed to restore levels of ATP or creatine phosphate, maintain the redox balance of adenine nucleotides, or avert the oxidation of glutathione. Under the specified experimental parameters, EGT did not alleviate myocardial injury or oxidative stress subsequent to ischemia–reperfusion (91). Across these investigations, EGT exhibits context-dependent molecular effects primarily focused on the modulation of redox states and inflammatory signaling pathways. In vascular endothelial cells, EGT diminishes the accumulation of ROS and nitric oxide, indicating a partial reduction in oxidative stress and macrophage activation; however, this occurs in the absence of systemic metabolic enhancement in obese murine models. In the context of myocardial infarction, EGT provides enhanced cardioprotective effects by inhibiting NF-κB activation, downregulating Wnt5a and sFlt-1, and attenuating GLRX-associated s-glutathionylation, thereby mitigating inflammation, fibrosis, and the apoptosis of cardiomyocytes. Nevertheless, in the scenario of isolated ischemia–reperfusion subjected hearts, EGT does not succeed in maintaining the thiol/disulfide equilibrium, glutathione redox state, or energy metabolic processes, thereby signifying its limited capacity for direct protective effects against acute oxidative damage in this particular context.

**Table 5:** The effect of cytoprotective antioxidant of ergothioneine in cardiometabolic disorders.

Experimental model	Molecular targets/pathway	Effects on oxidative stress and inflammation	Effects on cardiac/vascular function	References
*In vitro* endothelial cells and macrophages under high-lipid conditions; diet-induced obese mice	ROS, nitric oxide (NO)	↓ ROS in macrophages; ↓ NO in endothelial cells; no effect on endothelial tube formation	No effect on circulating lipids, lipoproteins, or glucose in obese mice	(89)
Rat myocardial infarction model; HCAEC hypoxia model	NF-κB (p65, p-p65), Wnt5a, sFlt-1, GLRX, MCP-1; S-glutathionylation pathway	↓ MCP-1; ↓ NF-κB activation; ↓ sFlt-1; ↓ GLRX; reduced inflammatory signaling	Improved echocardiographic function; ↓ infarct size; ↓ fibrosis; ↓ cardiomyocyte death	(90)
Langendorff-perfused rabbit hearts (45 min ischemia + 30 min reperfusion)	Thiol/disulfide balance; GSH/GSSG; energy metabolism	No preservation of GSH; no reduction in GSSG; no improvement in redox balance	No improvement in developed pressure, diastolic pressure, CK/lactate release, ATP or CP levels	(91)

ROS, reactive oxygen species; NO, nitric oxide; NF-κB, nuclear factor kappa B; p65, NF-κB p65 subunit; p-p65, phosphorylated p65; Wnt5a, Wnt family member 5A; sFlt-1, soluble fms-like tyrosine kinase-1; GLRX, glutaredoxin-1; MCP-1, monocyte chemoattractant protein-1; HCAEC, human coronary artery endothelial cells; GSH, reduced glutathione; GSSG, oxidized glutathione; CK, creatine kinase; ATP, adenosine triphosphate; CP, creatine phosphate.

Cell-based studies provide mechanistic insights into EGT’s protective actions. In endothelial cells, cardiomyocytes, and neuronal models exposed to oxidative stressors, EGT consistently reduces reactive oxygen species (ROS) accumulation and protects mitochondrial membrane potential. Experimental approaches such as ROS fluorescence assays, lipid peroxidation measurements, and mitochondrial potential analyses demonstrate that EGT effectively limits oxidative damage at both the cellular and mitochondrial levels. In contrast to redox-cycling antioxidants such as alpha-lipoic acid, EGT acts as a stable scavenger of reactive oxygen and nitrogen species while also chelating redox-active metal ions, particularly iron and copper. This metal-chelating capacity is particularly relevant for preventing iron-dependent lipid peroxidation and ferroptotic cell death, processes increasingly implicated in cardiometabolic pathology. Animal studies further support the cytoprotective and anti-inflammatory properties of EGT. In rodent models of cardiovascular injury, metabolic syndrome, and oxidative stress, EGT supplementation has been associated with reductions in lipid peroxidation markers, improved antioxidant enzyme activity, and preservation of mitochondrial ultrastructure. Methodologically, these investigations often combine biochemical assays of oxidative damage with histological analysis and electron microscopy to evaluate mitochondrial morphology. Some studies also report attenuation of inflammatory signaling pathways, including reductions in pro-inflammatory cytokines and improved endothelial function. Compared with research on ALA or UA, however, the number of animal studies on EGT remains relatively limited, reflecting the relatively recent recognition of its physiological importance.

Human evidence supporting the cardiometabolic relevance of EGT is currently dominated by observational and epidemiological studies. Several population-based analyses have reported associations between higher circulating EGT levels and reduced risk of cardiovascular disease, cognitive decline, and all-cause mortality. These studies typically rely on plasma metabolomic profiling and long-term cohort analyses to identify correlations between EGT status and health outcomes. Although such findings cannot establish causality, they provide compelling evidence that EGT may serve as both a protective dietary factor and a biomarker of reduced oxidative stress burden. Taken together, the available evidence positions EGT as an emerging cytoprotective antioxidant with potential relevance for cardiometabolic disease prevention. Its unique biochemical stability, targeted tissue uptake, and capacity to limit oxidative and metal-mediated damage distinguish it from many conventional antioxidants. Nevertheless, the current evidence base remains less developed than that for ALA or UA, highlighting the need for controlled clinical trials to clarify its therapeutic efficacy and optimal supplementation strategies.

## 7 Comparative mechanistic insights

**Figure 2:**
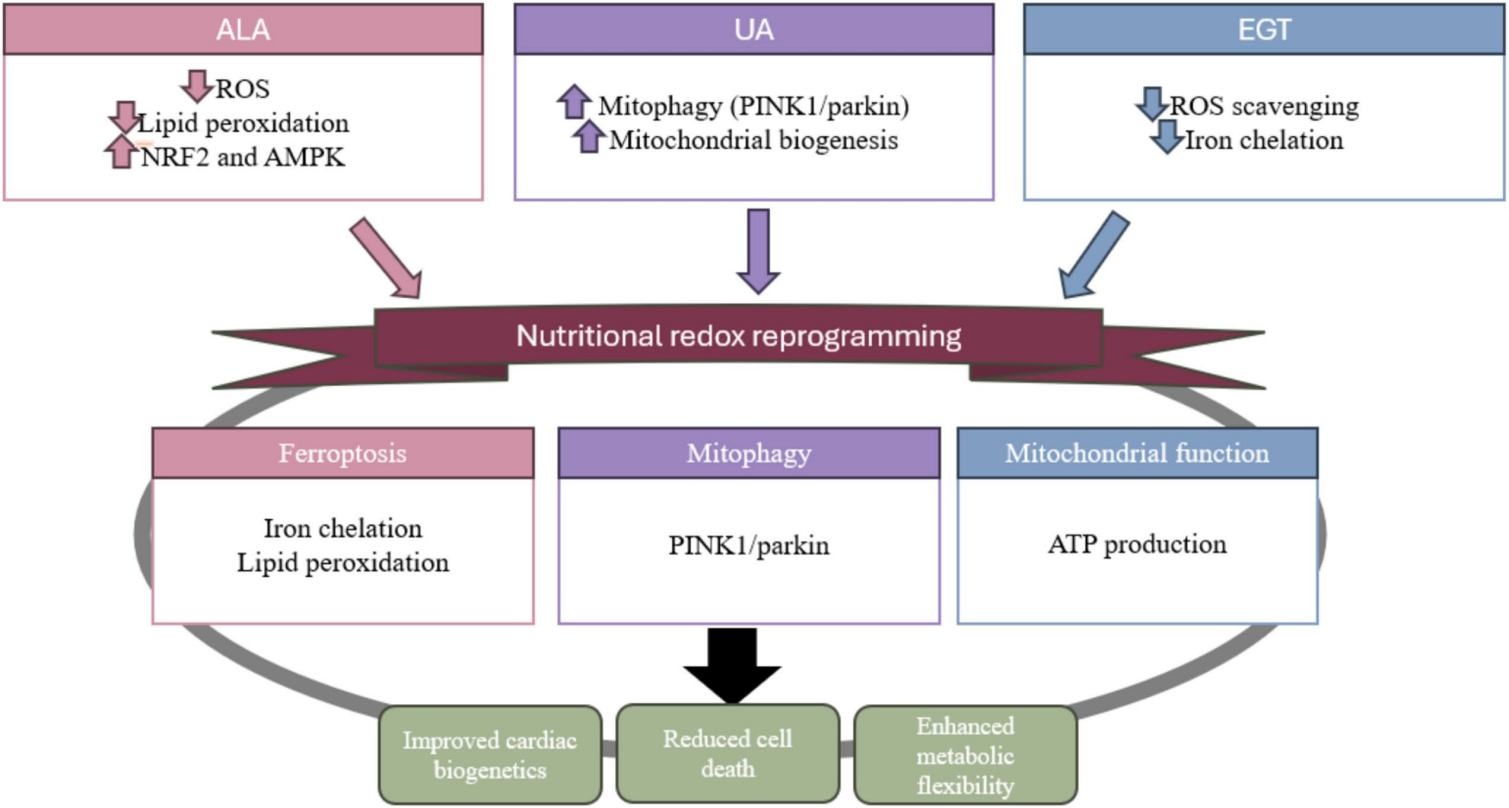
Integrated model of nutritional redox reprogramming in cardiometabolic disease. This schematic illustrates the convergent and complementary roles of alpha-lipoic acid (ALA), urolithin A (UA), and ergothioneine (EGT) in modulating key pathological processes underlying cardiometabolic disorders. ALA primarily regulates redox homeostasis by reducing reactive oxygen species (ROS), inhibiting lipid peroxidation, and activating antioxidant signaling pathways such as Nrf2 and AMPK, thereby suppressing ferroptotic signaling. UA predominantly enhances mitochondrial quality control by activating PINK1/Parkin-mediated mitophagy and promoting mitochondrial biogenesis through PGC-1α, facilitating the removal of dysfunctional mitochondria and improving metabolic efficiency. EGT acts as a stable thiol antioxidant that scavenges ROS, chelates redox-active iron, and protects mitochondrial integrity, thereby limiting iron-dependent lipid peroxidation and ferroptosis initiation. These compounds converge on a central framework of “nutritional redox reprogramming,” integrating the regulation of ferroptosis, mitophagy, and mitochondrial function. Ferroptosis is characterized by iron accumulation and lipid peroxidation, mitophagy ensures mitochondrial quality control through selective autophagic removal of damaged mitochondria, and mitochondrial function governs ATP production and cellular bioenergetics. Through coordinated modulation of these interconnected pathways, ALA, UA, and EGT collectively reduce cardiomyocyte death, enhance cardiac bioenergetics, and improve metabolic flexibility, ultimately contributing to cardioprotection in conditions such as atherosclerosis and myocardial ischemia.

Instead of acting as traditional antioxidants with redundant functions, ALA, UA, and EGT appear to modulate partly distinct yet interconnected components of the ferroptosis–mitophagy–mitochondrial metabolism regulatory network. Evidence drawn from experimental cardiometabolic studies indicates that these compounds jointly affect susceptibility to lipid peroxidation, regulation of mitochondrial quality control pathways, and metabolic adaptability through complementary biological mechanisms. Therefore, their cardioprotective effects are more accurately understood within an integrated redox systems perspective, rather than as the independent actions of individual antioxidant agents (Figure 2).

### 7.1 Ferroptosis modulation: similarities and differences

ALA, UA, and EGT each demonstrate modulation of ferroptosis, albeit through distinct molecular pathways. Research indicates that UA has the capacity to mitigate ferroptotic cell death in ischemia–reperfusion scenarios by promoting Nrf2 nuclear translocation and activating downstream antioxidant gene expression, thereby curtailing lipid peroxidation and iron-dependent ROS production (80). ALA, traditionally recognized for its antioxidant properties, indirectly attenuates ferroptotic signaling by enhancing glutathione concentrations and Nrf2 activity, which serves to inhibit iron-catalyzed lipid radical formation (92). EGT, characterized as a thiol-based antioxidant, effectively scavenges lipid peroxides and stabilizes iron–sulfur clusters, thereby exhibiting protective effects against ferroptosis in both cardiomyocytes and vascular endothelial cells (93). In comparison, the inhibition of ferroptosis by UA appears to be most directly associated with transcriptional reprogramming, while ALA and EGT predominantly fortify redox buffering mechanisms.

A further dimension of mechanistic differentiation among these compounds emerges when evaluating their effects on iron transport systems and ferroptosis-modulating antioxidant enzymes that set the threshold for lipid peroxide propagation. In addition to facilitating glutathione recycling, ALA has been reported to regulate cellular iron balance by influencing both iron uptake and export processes. Specifically, it suppresses DMT1 expression while increasing ferroportin and ferritin heavy chain levels, thereby lowering the intracellular labile iron pool and reducing Fenton reaction–mediated lipid radical generation. At the same time, ALA enhances GPX4 activity via NRF2-dependent pathways, strengthening phospholipid hydroperoxide detoxification and supporting mitochondrial membrane stability under oxidative stress. Together, these actions establish ALA as an indirect yet functionally significant modulator of ferroptosis susceptibility at the convergence of glutathione metabolism and iron handling.

In contrast, UA appears to inhibit ferroptotic signaling mainly through transcriptional activation of endogenous cytoprotective antioxidant pathways rather than through direct regulation of intracellular iron stores. In cardiometabolic ischemia–reperfusion models, UA has been shown to stimulate Nrf2 nuclear translocation and induce downstream effectors such as HO-1, thereby reducing mitochondrial ROS accumulation and lipid peroxidation while maintaining cardiomyocyte survival. This gene-level enhancement of intrinsic antioxidant defenses indicates that UA acts upstream of lipid peroxidation execution by reshaping stress-responsive transcriptional networks that determine ferroptotic sensitivity.

EGT, by comparison, operates through a distinct mechanism as a mitochondria-directed thiol antioxidant with strong affinity for redox-active transition metals. Its uptake via the OCTN1 (SLC22A4) transporter enables selective intracellular accumulation within mitochondria and regions of oxidative damage. In these compartments, EGT helps preserve iron–sulfur cluster integrity, buffer labile iron pools, and directly scavenge lipid-derived peroxyl radicals. Through this combination of metal chelation and radical-neutralizing activity, EGT is particularly suited to disrupt early ferroptotic initiation processes driven by mitochondrial ROS amplification and iron-dependent phospholipid oxidation in cardiomyocytes and vascular endothelial cells.

Collectively, these findings indicate that ALA, UA, and EGT exert anti-ferroptotic effects at partially distinct regulatory points along the ferroptosis cascade. UA primarily strengthens transcriptional antioxidant defense programs; ALA integrates glutathione-dependent peroxide detoxification with control of iron transport pathways; and EGT directly stabilizes mitochondrial redox-active iron and modulates lipid radical propagation dynamics. This complementary distribution of activity, spanning upstream transcriptional regulation, intermediary antioxidant buffering, and downstream lipid peroxide propagation, supports the rationale that simultaneous targeting of these ferroptosis-relevant nodes may offer a mechanistically synergistic approach to mitigating cardiometabolic ferroptotic injury.

### 7.2 Mitophagy induction capacity

Mitophagy serves to eliminate impaired mitochondria, thereby sustaining cellular homeostasis. UA initiates mitophagy through the Nrf2–p62 signaling pathway, thereby enhancing mitochondrial quality following cellular injury (94). ALA promotes mitophagic flux via Parkin-dependent mechanisms, thereby safeguarding mitochondrial integrity in the context of oxidative stress (95). EGT facilitates mitophagy indirectly by preserving mitochondrial redox equilibrium (96). UA presents the most compelling evidence for the induction of targeted mitophagy in the context of cardiometabolic stress.

A further point of mechanistic differentiation becomes evident when the actions of these compounds are viewed within the hierarchical organization of mitochondrial quality-control networks rather than as isolated triggers of mitophagy. Among them, UA most consistently exhibits direct activation of mitophagic machinery through coordinated engagement of the Nrf2–p62 signaling axis and subsequent recruitment of Parkin-dependent mitochondrial clearance pathways. In ischemia–reperfusion and metabolic injury models referenced in this manuscript, UA-induced increases in mitophagic flux correlate with recovery of mitochondrial membrane potential, attenuation of mitochondrial ROS amplification, and improved ATP production. These findings support its role as an upstream regulator of mitochondrial turnover, extending beyond simple redox stabilization. By contrast, ALA appears to promote mitophagy chiefly by sustaining the redox conditions necessary for effective mitochondrial quality-control signaling rather than by directly initiating mitophagic cascades. Experimental studies indicate that ALA facilitates Parkin translocation to damaged mitochondria under oxidative stress while preserving glutathione-dependent antioxidant capacity and limiting lipid peroxide accumulation that might otherwise disrupt mitophagy progression. Through these integrated actions, ALA enhances completion of mitophagic flux and prevents the persistence of dysfunctional mitochondria that drive ferroptosis-prone metabolic remodeling in cardiometabolic tissues.

EGT, in comparison, influences mitophagy primarily through maintenance of mitochondrial redox balance and preservation of iron–sulfur cluster stability rather than through direct stimulation of canonical mitophagy pathways. Its preferential intracellular uptake via the OCTN1 transporter enables mitochondrial accumulation at sites of oxidative stress, where it dampens ROS generation and mitigates mitochondrial injury that would otherwise provoke compensatory mitophagic activation. This pattern suggests that EGT acts principally as a mitochondrial protective agent, indirectly supporting mitophagy efficiency by maintaining structural and metabolic integrity under cardiometabolic stress. Collectively, these compounds appear to function at distinct yet complementary checkpoints along the mitophagy continuum. UA predominantly enhances the initiation of selective mitochondrial clearance; ALA supports the propagation and successful completion of mitophagic flux during oxidative stress; and EGT safeguards mitochondrial integrity upstream of mitophagy activation by limiting redox-driven damage. This functional stratification reinforces the concept that nutritionally derived redox modulators regulate mitochondrial quality control through coordinated but mechanistically differentiated pathways that together strengthen mitochondrial resilience in cardiometabolic disease.

Emerging data further indicate that improved mitophagic efficiency mediated by these agents may indirectly suppress ferroptotic signaling by curbing mitochondrial ROS amplification and subsequent lipid peroxidation. Within this integrated model, UA-driven mitophagy initiation, ALA-supported flux completion, and EGT-mediated mitochondrial stabilization converge to modulate the ferroptosis–mitophagy regulatory axis in cardiometabolic pathology through complementary mechanisms.

### 7.3 Effects on mitochondrial respiration and metabolic flexibility

UA reinstates the mitochondrial membrane potential and ATP synthesis, thereby restoring fatty acid oxidation processes within ischemic myocardial tissue (80). ALA functions as a crucial cofactor for mitochondrial dehydrogenases, thereby augmenting the efficiency of the electron transport chain and mitigating ROS leakage (97). EGT is sequestered within mitochondria through organic cation transporters, thereby preserving redox equilibrium and enhancing respiratory efficiency (24). UA promotes metabolic reprogramming subsequent to injury, while ALA and EGT predominantly contribute to the maintenance of basal bioenergetic resilience.

A clearer mechanistic distinction among these compounds emerges when their effects are interpreted within the framework of mitochondrial metabolic flexibility rather than static measurements of respiratory efficiency alone. UA consistently demonstrates the strongest capacity to promote adaptive metabolic reprogramming following cardiometabolic stress by restoring mitochondrial membrane potential, enhancing oxidative phosphorylation efficiency, and facilitating recovery of fatty acid β-oxidation pathways in ischemic myocardial tissue. Across the experimental models discussed in this manuscript, these effects are closely associated with activation of mitochondrial quality-control signaling and improved coupling between substrate utilization and ATP production, supporting the interpretation that UA functions primarily as a regulator of injury-responsive metabolic remodeling rather than a basal respiratory cofactor.

In contrast, ALA exerts its principal effects on mitochondrial metabolism through its established role as an essential cofactor for mitochondrial α-ketoacid dehydrogenase complexes, including pyruvate dehydrogenase and α-ketoglutarate dehydrogenase, thereby facilitating efficient entry of glycolytic substrates into the tricarboxylic acid cycle and sustaining electron transport chain activity under oxidative stress conditions. In addition to enhancing substrate flux through central carbon metabolism, ALA-mediated regeneration of reduced glutathione limits mitochondrial ROS accumulation and preserves respiratory chain integrity, thereby maintaining redox-sensitive enzymatic activity required for sustained ATP synthesis in cardiometabolic tissues. These combined effects position ALA as a stabilizer of basal mitochondrial bioenergetic competence rather than a primary inducer of metabolic reprogramming following injury.

EGT differs mechanistically from both UA and ALA by exerting its metabolic effects predominantly through preservation of mitochondrial redox homeostasis and protection of iron–sulfur cluster–containing respiratory enzymes that are particularly vulnerable to oxidative disruption. Preferential intracellular accumulation through OCTN1-dependent transport enables EGT to localize within mitochondria and buffer ROS at sites of high metabolic flux, thereby supporting maintenance of electron transport chain efficiency and limiting oxidative impairment of complex I and complex III activity. Through stabilization of these redox-sensitive components of mitochondrial respiration, EGT appears to preserve metabolic resilience under conditions of cardiometabolic stress rather than directly promoting substrate-driven metabolic switching.

Importantly, these compounds appear to operate at complementary regulatory levels within the continuum of mitochondrial metabolic adaptation. UA primarily supports injury-responsive metabolic reprogramming through restoration of oxidative phosphorylation capacity and fatty acid utilization pathways, ALA maintains central carbon metabolic flux and enzymatic efficiency within the tricarboxylic acid cycle, and EGT preserves respiratory chain structural integrity through targeted mitochondrial redox buffering. This functional stratification suggests that nutritionally derived redox modulators collectively enhance mitochondrial metabolic flexibility by coordinating substrate utilization efficiency, respiratory chain stability, and ROS-dependent signaling thresholds that influence both mitophagy activation and ferroptosis susceptibility in cardiometabolic disease contexts.

Emerging evidence further indicates that preservation of mitochondrial metabolic flexibility by these compounds may indirectly suppress ferroptotic signaling by limiting mitochondrial ROS amplification and phospholipid peroxidation propagation while simultaneously reducing the burden of dysfunctional mitochondria requiring mitophagic clearance. Within this integrated framework, coordinated regulation of mitochondrial respiration by UA, ALA, and EGT represents a central mechanistic axis linking metabolic adaptation to redox-sensitive cardiometabolic protection.

### 7.4 Translational strength of current evidence

ALA possesses the most comprehensive clinical evidence, demonstrating improvements in systemic inflammation and endothelial function among cardiometabolic patients (98). EGT display encouraging preclinical cardiometabolic effects but are devoid of clinical trial data (99). As a result, ALA presents the most robust translational evidence, followed by UA, while ET emerges as a potential candidate.

A more coherent view of the translational landscape emerges when these compounds are classified by their mechanistic roles within mitochondrial redox regulation rather than by the sheer volume of clinical studies available. Among the three agents discussed, ALA currently shows the greatest degree of clinical preparedness. This is largely attributable to its established biochemical function as a mitochondrial cofactor and facilitator of glutathione recycling, which enables relatively predictable modulation of systemic oxidative stress and endothelial dysfunction across diverse cardiometabolic populations. Reported clinical improvements in inflammatory biomarkers, vascular responsiveness, and insulin sensitivity align with experimental findings demonstrating ALA’s capacity to preserve mitochondrial bioenergetics and attenuate lipid peroxidation–related injury pathways linked to ferroptotic vulnerability.

UA, by comparison, occupies a transitional translational stage. Although preclinical cardiometabolic models provide robust mechanistic evidence supporting its effects, controlled human data remain comparatively limited. Experimental studies consistently show that UA enhances mitophagic flux, restores mitochondrial membrane potential, and improves oxidative phosphorylation efficiency following ischemic or metabolic stress. However, because UA is generated through gut microbiota–mediated metabolism of dietary ellagitannins, variability in microbial composition across individuals may significantly influence systemic exposure and therapeutic response. This microbiome-dependent activation introduces an important translational variable when considering reproducibility in heterogeneous cardiometabolic populations.

EGT represents an earlier-stage translational candidate with strong mechanistic rationale but limited large-scale clinical validation. Its therapeutic promise stems from distinctive intracellular transport characteristics and mitochondria-targeted antioxidant properties. Uptake via the OCTN1 transporter facilitates selective tissue accumulation in environments characterized by elevated oxidative stress, such as vascular endothelium and cardiomyocytes. Within these settings, EGT contributes to stabilization of iron–sulfur cluster–containing enzymes and buffering of redox-active transition metals implicated in ferroptotic lipid peroxidation cascades. Although observational and experimental findings suggest a cardiometabolic protective role, rigorously controlled intervention trials examining mitochondrial functional outcomes and ferroptosis-associated lipid peroxidation biomarkers remain sparse.

Importantly, differences in translational maturity likely reflect not only disparities in clinical trial volume but also variation in the availability of measurable pharmacodynamic markers that directly link mitochondrial redox modulation to cardiometabolic outcomes. For ALA, systemic oxidative stress indices and endothelial function assessments provide accessible clinical endpoints. In contrast, effective translation of UA-driven mitophagy enhancement and EGT-mediated iron buffering will require integration of emerging mitochondrial biomarkers, such as lipid peroxidation signatures, mitophagy-related protein expression patterns, and iron-handling pathway indicators. Incorporating these mechanistically aligned measures into future trials could substantially enhance interpretability of nutritional modulation of the ferroptosis–mitophagy axis in cardiometabolic disease.

Overall, current evidence positions ALA as the most immediately actionable mitochondrial redox modulator among the compounds reviewed, while UA and EGT remain compelling candidates for further translational development within precision nutrition frameworks targeting mitochondrial dysfunction in cardiometabolic pathology. A clinically informed, mechanism-based evaluation strategy incorporating mitochondrial quality-control metrics and ferroptosis-relevant lipid biomarkers may help clarify their therapeutic roles and guide future combination approaches.

## 8 Clinical translation and future directions

Despite encouraging preclinical findings, the clinical application of ALA, UA, and EGT encounters significant limitations pertaining to bioavailability. ALA demonstrates rapid absorption; however, it is also subject to swift hepatic metabolism and exhibits a brief plasma half-life, which may impede prolonged tissue exposure. The disparities between racemic mixtures and R-enantiomer formulations further exacerbate the complexities associated with dose standardization. The biosynthesis of UA is contingent upon the composition of the gut microbiota, leading to considerable inter-individual variability in plasma concentrations and therapeutic efficacy. The bioavailability of EGT is modulated by the expression and functionality of the OCTN1 (SLC22A4) transporter, which facilitates cellular uptake and tissue accumulation. Future research initiatives should prioritize the development of optimized formulations, targeted delivery mechanisms, and comprehensive pharmacokinetic profiling to guarantee adequate mitochondrial concentrations within cardiovascular tissues. Given the complex multifactorial etiology of cardiometabolic disorders, the implementation of combination strategies may enhance therapeutic effectiveness. The integration of redox-active nutraceuticals with established pharmacological interventions (such as statins, antiplatelet agents, or SGLT2 inhibitors) has the potential to yield additive or synergistic advantages by concurrently targeting complementary biological pathways, including the suppression of ferroptosis, the reduction of inflammation, and the optimization of mitochondrial bioenergetics. Furthermore, the co-administration of ALA with mitophagy enhancers, such as UA, may significantly bolster mitochondrial quality control, whereas EGT may enhance antioxidant buffering capabilities. Systematic exploration of rational multi-target approaches is warranted within translational research frameworks. The observed inter-individual variability in redox status, mitochondrial functionality, metabolic adaptability, and the composition of gut microbiota indicates that a universal “one-size-fits-all” strategy is unlikely to yield optimal outcomes. Precision nutrition paradigms that consider genetic polymorphisms, metabolomic profiles, inflammatory biomarkers, and microbiome analyses could facilitate the identification of cardiometabolic phenotypes most likely to derive benefits from specific redox modulators. Stratified methodologies may prove particularly beneficial for patients exhibiting increased susceptibility to ferroptosis, compromised mitophagy, or metabolic rigidity. While mechanistic and animal investigations present persuasive evidence, the availability of comprehensive randomized controlled trials (RCTs) remains insufficient. It is imperative to conduct large-scale, methodologically rigorous RCTs that incorporate standardized dosing regimens, explicitly defined cardiovascular endpoints, and assessments of mechanistic biomarkers to ascertain clinical efficacy. Subsequent trials ought to integrate evaluations of ferroptosis markers, indices of mitochondrial respiration, biomarkers of oxidative stress, and parameters of cardiac function assessed through imaging techniques. Furthermore, the long-term safety profile, interactions between drugs and nutrients, and responses specific to diverse populations must be meticulously investigated prior to the incorporation of these compounds into evidence-based cardiometabolic management.

## 9 Conclusion

Cardiometabolic diseases are increasingly acknowledged as conditions characterized by redox imbalance, mitochondrial dysfunction, and maladaptive signaling pathways leading to cell death, including ferroptosis. The intersection of oxidative stress, impaired mitophagy, and disrupted mitochondrial metabolism establishes a comprehensive mechanistic framework that connects atherosclerosis, MI, and myocardial infarction. In this regard, nutritional redox reprogramming emerges as an innovative therapeutic paradigm shift that transcends traditional antioxidant supplementation, focusing instead on the targeted modulation of redox-sensitive signaling networks and the systems governing mitochondrial quality control. ALA, UA, and EGT serve as quintessential representatives of an emerging class of bioactive compounds that possess the capacity to modulate interrelated pathways that regulate susceptibility to ferroptosis, mitophagic flux, and cardiac bioenergetics. Instead of merely functioning as radical scavengers, these compounds seem to operate as facilitators of cellular adaptation, metabolic versatility, and resilience against stressors. Their multifaceted actions underscore the promise of nutritionally sourced molecules to transform mitochondrial functionality and reestablish redox equilibrium in cardiometabolic tissues. Although challenges in translation persist, the amalgamation of mechanistic understanding with precision nutrition paradigms and comprehensive clinical assessments may foster the creation of tailored therapeutic interventions. Ultimately, nutritional redox reprogramming embodies a progressive strategy aimed at both the prevention and mitigation of cardiovascular disease progression by directly addressing the mitochondrial–redox axis at its fundamental level.

Overall, the current body of evidence suggests that ALA, UA, and EGT represent a promising group of nutritionally derived compounds that converge on mitochondrial protection and redox homeostasis through complementary mechanisms. ALA primarily acts as a multifunctional redox modulator and mitochondrial cofactor, UA promotes mitochondrial quality control through mitophagy and mitochondrial biogenesis, and EGT functions as a stable cytoprotective antioxidant with selective cellular uptake. Together, these mechanisms address key pathological processes underlying cardiometabolic disorders, including oxidative stress, mitochondrial dysfunction, and impaired cellular energy metabolism. The reliability of the available data is moderate but uneven across compounds: ALA is supported by extensive experimental and clinical studies, UA by strong mechanistic and animal evidence with emerging human trials, and EGT mainly by cellular research and epidemiological observations. Consequently, while the collective findings provide a biologically coherent and increasingly well-supported framework, further well-designed long-term clinical studies are required to confirm the translational significance of these compounds for cardiovascular prevention and therapy.

In interpreting the available evidence, it is important to note that much of the current knowledge regarding ALA, UA, and EGT is derived from cellular and animal studies that provide valuable mechanistic insights into their roles in redox regulation, mitochondrial function, and cellular protection. While these experimental findings consistently support the biological plausibility of these compounds in modulating pathways relevant to cardiometabolic disorders, the amount of clinical and translational evidence remains comparatively limited, particularly for UA and EGT. ALA currently has the strongest clinical support, whereas human studies investigating the physiological and therapeutic effects of UA and EGT are still emerging. Therefore, although the existing data collectively suggest promising protective roles for these compounds, further well-designed clinical trials are required to confirm their efficacy, determine optimal dosing strategies, and clarify their long-term impact on cardiovascular and metabolic health.

## References

[1] Tan SCW Zheng BB Tang ML Chu H Zhao YT Weng C. Global burden of cardiovascular diseases and its risk factors, 1990-2021: a systematic analysis for the global burden of disease study 2021. Q J Med. (2025) 118:411–22. doi: 10.1093/qjmed/hcaf022, 39847534

[2] Madamanchi NR Runge MS. Mitochondrial dysfunction in atherosclerosis. Circ Res. (2007) 100:460–73. doi: 10.1161/01.res.0000258450.44413.96, 17332437

[3] Murphy MP. How mitochondria produce reactive oxygen species. Biochem J. (2009) 417:1–13. doi: 10.1042/BJ20081386 19061483 PMC2605959

[4] Dorn GW 2nd Kitsis RN. The mitochondrial dynamism-mitophagy-cell death interactome: multiple roles performed by members of a mitochondrial molecular ensemble. Circ Res. (2015) 116:167–82. doi: 10.1161/CIRCRESAHA.116.303554, 25323859 PMC4282600

[5] Dixon SJ Lemberg KM Lamprecht MR Skouta R Zaitsev EM Gleason CE . Ferroptosis: an iron-dependent form of nonapoptotic cell death. Cell. (2012) 149:1060–72. doi: 10.1016/j.cell.2012.03.042, 22632970 PMC3367386

[6] Fang X Wang H Han D Xie E Yang X Wei J . Ferroptosis as a target for protection against cardiomyopathy. Proc Natl Acad Sci USA. (2019) 116:2672–80. doi: 10.1073/pnas.1821022116, 30692261 PMC6377499

[7] Halliwell B. The antioxidant paradox: less paradoxical now? Br J Clin Pharmacol. (2013) 75:637–44. doi: 10.1111/j.1365-2125.2012.04272.x, 22420826 PMC3575931

[8] Shay KP Moreau RF Smith EJ Smith AR Hagen TM. Alpha-lipoic acid as a dietary supplement: molecular mechanisms and therapeutic potential. Biochim Biophys Acta. (2009) 1790:1149–60. doi: 10.1016/j.bbagen.2009.07.026, 19664690 PMC2756298

[9] Ryu D Mouchiroud L Andreux PA Katsyuba E Moullan N Nicolet-Dit-Félix AA . Urolithin A induces mitophagy and prolongs lifespan in *C. elegans* and increases muscle function in rodents. Nat Med. (2016) 22:27400265:879–88. doi: 10.1038/nm.4132 27400265

[10] Cheah IK Halliwell B. Ergothioneine; antioxidant potential, physiological function and role in disease. Biochim Biophys Acta. (2012) 1822:784–93. doi: 10.1016/j.bbadis.2011.09.017, 22001064

[11] Khan H Singh TG Dahiya RS Abdel-Daim MM. α-Lipoic acid, an organosulfur biomolecule a novel therapeutic agent for neurodegenerative disorders: an mechanistic perspective. Neurochem Res. (2022) 47:1853–64. doi: 10.1007/s11064-022-03598-w, 35445914

[12] Solmonson A DeBerardinis RJ. Lipoic acid metabolism and mitochondrial redox regulation. J Biol Chem. (2018) 293:7522–30. doi: 10.1074/jbc.TM117.000259, 29191830 PMC5961061

[13] Patel MS Packer L. Lipoic acid: Energy Production, Antioxidant Activity and Health Effects. Boca Raton, Florida, USA: CRC Press (2008).

[14] Dieter F Esselun C Eckert GP. Redox active α-lipoic acid differentially improves mitochondrial dysfunction in a cellular model of Alzheimer and its control cells. Int J Mol Sci. (2022) 23:9186. doi: 10.3390/ijms23169186, 36012451 PMC9409376

[15] Ghibu S Richard C Vergely C Zeller M Cottin Y Rochette L. Antioxidant properties of an endogenous thiol: alpha-lipoic acid, useful in the prevention of cardiovascular diseases. J Cardiovasc Pharmacol. (2009) 54:391–8. doi: 10.1097/FJC.0b013e3181be7554, 19998523

[16] Cerdá B Periago P Espín JC Tomás-Barberán FA. Identification of urolithin a as a metabolite produced by human colon microflora from ellagic acid and related compounds. J Agric Food Chem. (2005) 53:5571–6. doi: 10.1021/jf050384i, 15998116

[17] Aichinger G. Natural dibenzo-α-pyrones: friends or foes? Int J Mol Sci. (2021) 22:13063. doi: 10.3390/ijms222313063, 34884865 PMC8657677

[18] Zhang Q Zhang W Yuan X Peng X Hu G. Urolithin A in central nervous system disorders: therapeutic applications and challenges. Biomedicine. (2025) 13:1553. doi: 10.3390/biomedicines13071553, 40722629 PMC12292995

[19] Song H Yun C Choi YJ Jeong W Kim Y Kim J . Urolithin A restores mitochondrial function and reverses cardiac remodeling in heart failure with preserved ejection fraction. bioRxiv. (2025). doi: 10.64898/2025.12.26.696570

[20] Esselun C Theyssen E Eckert GP. Effects of urolithin A on mitochondrial parameters in a cellular model of early Alzheimer disease. Int J Mol Sci. (2021) 22:8333. doi: 10.3390/ijms22158333, 34361099 PMC8347929

[21] Liu S Faitg J Tissot C Konstantopoulos D Laws R Bourdier G . Urolithin A provides cardioprotection and mitochondrial quality enhancement preclinically and improves human cardiovascular health biomarkers. Iscience. (2025) 28:111814. doi: 10.1016/j.isci.2025.111814 40034121 PMC11875685

[22] Ey J Schömig E Taubert D. Dietary sources and antioxidant effects of ergothioneine. J Agric Food Chem. (2007) 55:6466–74. doi: 10.1021/jf071328f 17616140

[23] Halliwell B Cheah IK Tang RM. Ergothioneine–a diet-derived antioxidant with therapeutic potential. FEBS Lett. (2018) 592:3357–66. doi: 10.1002/1873-3468.13123, 29851075

[24] Borodina I Kenny LC McCarthy CM Paramasivan K Pretorius E Roberts TJ . The biology of ergothioneine, an antioxidant nutraceutical. Nutr Res Rev. (2020) 33:190–217. doi: 10.1017/S0954422419000301, 32051057 PMC7653990

[25] Cheah IK Halliwell B. Ergothioneine, recent developments. Redox Biol. (2021) 42:101868. doi: 10.1016/j.redox.2021.101868, 33558182 PMC8113028

[26] Smith E Ottosson F Hellstrand S Ericson U Orho-Melander M Fernandez C . Ergothioneine is associated with reduced mortality and decreased risk of cardiovascular disease. Heart. (2020) 106:691–7. doi: 10.1136/heartjnl-2019-315485, 31672783 PMC7229907

[27] Fu T-T Shen L. Ergothioneine as a natural antioxidant against oxidative stress-related diseases. Front Pharmacol. (2022) 13:850813. doi: 10.3389/fphar.2022.850813, 35370675 PMC8971627

[28] Obayashi K Kurihara K Okano Y Masaki H Yarosh DB. L-Ergothioneine scavenges superoxide and singlet oxygen and suppresses TNF-α and MMP-1 expression in UV-irradiated human dermal fibroblasts. Int J Cosmet Sci. (2005) 27:191. doi: 10.1111/j.0142-5463.2005.00265 15744438

[29] Servillo L D'Onofrio N Balestrieri ML. Ergothioneine antioxidant function: from chemistry to cardiovascular therapeutic potential. J Cardiovasc Pharmacol. (2017) 69:183–91. doi: 10.1097/FJC.0000000000000464, 28375902

[30] Wang K Dong Y Liu J Qian L Wang T Gao X . Effects of REDOX in regulating and treatment of metabolic and inflammatory cardiovascular diseases. Oxidative Med Cell Longev. (2020) 2020:5860356. doi: 10.1155/2020/5860356, 33282111 PMC7685846

[31] Penna C Pagliaro P. Endothelial dysfunction: redox imbalance, NLRP3 inflammasome, and inflammatory responses in cardiovascular diseases. Antioxidants. (2025) 14:256. doi: 10.3390/antiox14030256, 40227195 PMC11939635

[32] Balakrishnan VK Rajkumar A Ganesh MK Kovvuri HR Selvam D Krishnamurthy P . Redox imbalance and genetic mutations in heart failure: synergistic mechanisms and therapeutic strategies. Genes. (2026) 17:225. doi: 10.3390/genes17020225 41751609 PMC12940293

[33] Kattoor AJ Pothineni NVK Palagiri D Mehta JL. Oxidative stress in atherosclerosis. Curr Atheroscler Rep. (2017) 19:42. doi: 10.1007/s11883-017-0678-6 28921056

[34] De Rosa S Cirillo P Paglia A Sasso L Di Palma V Chiariello M. Reactive oxygen species and antioxidants in the pathophysiology of cardiovascular disease: does the actual knowledge justify a clinical approach? Curr Vasc Pharmacol. (2010) 8:259–75. doi: 10.2174/157016110790887009, 19758111

[35] Xu X Xu XD Ma MQ Liang Y Cai YB Zhu ZX . The mechanisms of ferroptosis and its role in atherosclerosis. Biomed Pharmacother. (2024) 171:116112. doi: 10.1016/j.biopha.2023.116112, 38171246

[36] Solanelles Curco À Garcia E Polishchuk A La Chica-Lhoëst MT Llorente-Cortes V. Impaired cholesterol and LDL uptake pathways in the development of oncological and cardiovascular diseases. Semin Cancer Biol. (2025) 116:84–95. doi: 10.1016/j.semcancer.2025.09.004, 40998097

[37] Laukaitiene J Gujyte G Kadusevicius E. Cardiomyocyte damage: ferroptosis relation to ischemia-reperfusion injury and future treatment options. Int J Mol Sci. (2023) 24:12846. doi: 10.3390/ijms241612846, 37629039 PMC10454599

[38] Wang B Wang Y Zhang J Hu C Jiang J Li Y . ROS-induced lipid peroxidation modulates cell death outcome: mechanisms behind apoptosis, autophagy, and ferroptosis. Arch Toxicol. (2023) 97:1439–51. doi: 10.1007/s00204-023-03476-6, 37127681

[39] Pervin M de Haan JB. Dysregulated redox signaling and its impact on inflammatory pathways, mitochondrial dysfunction, autophagy and cardiovascular diseases. Antioxidants (Basel, Switzerland). (2025) 14:1278. doi: 10.3390/antiox14111278 41300435 PMC12649175

[40] De R Mazumder S Bandyopadhyay U. Mediators of mitophagy that regulate mitochondrial quality control play crucial role in diverse pathophysiology. Cell Biol Toxicol. (2021) 37:333–66. doi: 10.1007/s10565-020-09561-1, 33067701

[41] Jiang M Xie X Cao F Wang Y. Mitochondrial metabolism in myocardial remodeling and mechanical unloading: implications for ischemic heart disease. Front Cardiovasc Med. (2021) 8:789267. doi: 10.3389/fcvm.2021.789267, 34957264 PMC8695728

[42] Perrelli MG Pagliaro P Penna C. Ischemia/reperfusion injury and cardioprotective mechanisms: role of mitochondria and reactive oxygen species. World J Cardiol. (2011) 3:186–200. doi: 10.4330/wjc.v3.i6.186, 21772945 PMC3139040

[43] Serrano JJ Medina M. Metabolic reprogramming at the edge of redox: connections between metabolic reprogramming and cancer redox state. Int J Mol Sci. (2025) 26:498. doi: 10.3390/ijms26020498, 39859211 PMC11765076

[44] Lai S Petramala L Muscaritoli M Cianci R Mazzaferro S Mitterhofer AP . α-Lipoic acid in patients with autosomal dominant polycystic kidney disease. Nutrition. (2020) 71:110594. doi: 10.1016/j.nut.2019.110594, 31790890

[45] Shen D Tian L Shen T Sun H Liu P. Alpha-lipoic acid protects human aortic endothelial cells against H2O2-induced injury and inhibits atherosclerosis in Ovariectomized low density lipoprotein receptor Knock-out mice. Cell Physiol Biochem. (2018) 47:2261–77. doi: 10.1159/000491537, 29975924

[46] Jang WG Kim HS Park KG Park YB Yoon KH Han SW . Analysis of proteome and transcriptome of tumor necrosis factor alpha stimulated vascular smooth muscle cells with or without alpha lipoic acid. Proteomics. (2004) 4:3383–93. doi: 10.1002/pmic.200400972 15378733

[47] Amom Z Zakaria Z Mohamed J Azlan A Bahari H Taufik Hidayat Baharuldin M . Lipid lowering effect of antioxidant alpha-lipoic acid in experimental atherosclerosis. J Clin Biochem Nutr. (2008) 43:88–94. doi: 10.3164/jcbn.2008051, 18818758 PMC2533724

[48] Lee WR Kim A Kim KS Park YY Park JH Kim KH . Alpha-lipoic acid attenuates atherosclerotic lesions and inhibits proliferation of vascular smooth muscle cells through targeting of the Ras/MEK/ERK signaling pathway. Mol Biol Rep. (2012) 39:6857–66. doi: 10.1007/s11033-012-1511-5, 22302393

[49] Yi X Maeda N. Alpha-lipoic acid prevents the increase in atherosclerosis induced by diabetes in apolipoprotein E-deficient mice fed high-fat/low-cholesterol diet. Diabetes. (2006) 55:2238–44. doi: 10.2337/db06-0251, 16873686

[50] Zulkhairi A Zaiton Z Jamaluddin M Sharida F Mohd TH Hasnah B . Alpha lipoic acid possess dual antioxidant and lipid lowering properties in atherosclerotic-induced New Zealand white rabbit. Biomed Pharmacother. (2008) 62:716–22. doi: 10.1016/j.biopha.2006.12.003 18538528

[51] Zhang WJ Bird KE McMillen TS LeBoeuf RC Hagen TM Frei B. Dietary alpha-lipoic acid supplementation inhibits atherosclerotic lesion development in apolipoprotein E-deficient and apolipoprotein E/low-density lipoprotein receptor-deficient mice. Circulation. (2008) 117:421–8. doi: 10.1161/CIRCULATIONAHA.107.725275 18158360

[52] Xu J Yang W Deng Q Huang Q Yang J Huang F. Flaxseed oil and α-lipoic acid combination reduces atherosclerosis risk factors in rats fed a high-fat diet. Lipids Health Dis. (2012) 11:148. doi: 10.1186/1476-511X-11-148, 23113997 PMC3502139

[53] Li X Zhang M Chen A Wang X Yang L Zhu Y . Lipoic acid nanoparticles exert effective Antiatherosclerosis effects through anti-inflammatory and antioxidant pathways. ACS Omega. (2024) 9:48642–9. doi: 10.1021/acsomega.4c07745, 39676958 PMC11635690

[54] Park KG Kim HS Ryu SY Nam CW Chae BK Dal Jung E . Alpha-lipoic acid inhibits TNF-alpha-induced fractalkine expression in rat aortic smooth muscle cells. Diabetes Metab J. (2005) 29:409–17.

[55] Ismawati I Asni E Mukhyarjon M Romus I. Alpha lipoic acid inhibits expression of intercellular adhesion molecule-1 (ICAM-1) in type 2 diabetic mellitus rat models. Indones Biomed J. (2020) 12:40–4. doi: 10.18585/inabj.v12i1.906

[56] BI AL-K Humadi AA Humadai TJ. Role of alpha lipoic acid effects on diabetic atherosclerosis in male rats: pathological and biochemical assay. Biochem Cell Arch. (2020) 20:25. doi: 10.35124/bca.2020.20.1.25

[57] Shama AM El-Bassiouny NA Bahnacy YE Werida RH. Impact of alpha lipoic acid as an adjuvant therapy on inflammation and fibrosis in type 2 diabetic patients with ischemic cardiomyopathy: a randomized controlled trial. J Diet Suppl. (2026) 23:1–22. doi: 10.1080/19390211.2025.2601020 41400270

[58] Oskuye ZZ Mehri K Mokhtari B Bafadam S Nemati S Badalzadeh R. Cardioprotective effect of antioxidant combination therapy: a highlight on MitoQ plus alpha-lipoic acid beneficial impact on myocardial ischemia-reperfusion injury in aged rats. Heliyon. (2024) 10:e28158. doi: 10.1016/j.heliyon.2024.e28158, 38524576 PMC10957437

[59] Qi B Zheng Y Gao W Qi Z Gong Y Liu Y . Alpha-lipoic acid impedes myocardial ischemia-reperfusion injury, myocardial apoptosis, and oxidative stress by regulating HMGB1 expression. Eur J Pharmacol. (2022) 933:175295. doi: 10.1016/j.ejphar.2022.175295, 36152839

[60] Gholami S Mokhtari B Badalzadeh R. Alpha-lipoic acid potentiates the anti-arrhythmic effects of ischemic postconditioning in the setting of cardiac ischemia/reperfusion injury in diabetic rats. J Diabetes Metab Disord. (2022) 21:707–16. doi: 10.1007/s40200-022-01034-y, 35673476 PMC9167407

[61] Mokhtari B Abdoli-Shadbad M Alihemmati A Javadi A Badalzadeh R. Alpha-lipoic acid preconditioning plus ischemic postconditioning provides additional protection against myocardial reperfusion injury of diabetic rats: modulation of autophagy and mitochondrial function. Mol Biol Rep. (2022) 49:1773–82. doi: 10.1007/s11033-021-06987-6 35098396

[62] Dudek M Knutelska J Bednarski M Nowiński L Zygmunt M Bilska-Wilkosz A . Alpha lipoic acid protects the heart against myocardial post ischemia-reperfusion arrhythmias via KATP channel activation in isolated rat hearts. Pharmacol Rep. (2014) 66:499–504. doi: 10.1016/j.pharep.2013.11.001, 24905530

[63] Cao X Chen A Yang P Song X Liu Y Li Z . Alpha-lipoic acid protects cardiomyocytes against hypoxia/reoxygenation injury by inhibiting autophagy. Biochem Biophys Res Commun. (2013) 441:935–40. doi: 10.1016/j.bbrc.2013.10.166, 24216106

[64] Deng C Sun Z Tong G Yi W Ma L Zhao B . α-Lipoic acid reduces infarct size and preserves cardiac function in rat myocardial ischemia/reperfusion injury through activation of PI3K/Akt/Nrf2 pathway. PLoS One. (2013) 8:e58371. doi: 10.1371/journal.pone.0058371, 23505496 PMC3591314

[65] He L Liu B Dai Z Zhang HF Zhang YS Luo XJ . Alpha lipoic acid protects heart against myocardial ischemia-reperfusion injury through a mechanism involving aldehyde dehydrogenase 2 activation. Eur J Pharmacol. (2012) 678:32–8. doi: 10.1016/j.ejphar.2011.12.042 22266491

[66] Wang X Yu Y Ji L Liang X Zhang T Hai CX. Alpha-lipoic acid protects against myocardial ischemia/reperfusion injury via multiple target effects. Food Chem Toxicol. (2011) 49:2750–7. doi: 10.1016/j.fct.2011.07.065 21843584

[67] Ko KM Yiu HY. Schisandrin B modulates the ischemia-reperfusion induced changes in non-enzymatic antioxidant levels in isolated-perfused rat hearts. Mol Cell Biochem. (2001) 220:141–7. doi: 10.1023/A:1010979404447, 11451374

[68] Coombes JS Powers SK Hamilton KL Demirel HA Shanely RA Zergeroglu MA . Improved cardiac performance after ischemia in aged rats supplemented with vitamin E and alpha-lipoic acid. Am J Physiol Regul Integr Comp Physiol. (2000) 279:R2149–55. doi: 10.1152/ajpregu.2000.279.6.R2149, 11080080

[69] Coombes JS Powers SK Demirel HA Jessup J Vincent HK Hamilton KL . Effect of combined supplementation with vitamin E and alpha-lipoic acid on myocardial performance during *in vivo* ischaemia-reperfusion. Acta Physiol Scand. (2000) 169:261–9. doi: 10.1046/j.1365-201x.2000.00740.x, 10951116

[70] Schönheit K Gille L Nohl H. Effect of alpha-lipoic acid and dihydrolipoic acid on ischemia/reperfusion injury of the heart and heart mitochondria. Biochim Biophys Acta. (1995) 1271:335–42. doi: 10.1016/0925-4439(95)00052-6, 7605800

[71] Wang Y Zheng Y Liang X Chang Y Liu Y Cheng X . Α-Lipoic acid alleviate myocardial infarction by suppressing age-independent macrophage senescence. Sci Rep. (2025) 15:11996. doi: 10.1038/s41598-025-92328-7, 40199978 PMC11978910

[72] Nemati S Zavvari-Oskuye Z Bafadam S Mokhtari B Badalzadeh R Vakili A. Impact of combined alpha-lipoic acid and mitoquinone supplementation on myocardial infarction in aged rats: heart performance and molecular mechanisms. Exp Gerontol. (2024) 189:112402. doi: 10.1016/j.exger.2024.112402 38484905

[73] Gholami S Badalzadeh R Alihemmati A. Alpha-lipoic acid enhances ischemic postconditioning-mediated improvement of myocardial infarction and apoptosis in diabetic rats with ischemia/reperfusion injury. Can J Physiol Pharmacol. (2023) 101:682–91. doi: 10.1139/cjpp-2023-0044, 37523770

[74] Wang Y Zheng Y Qi B Liu Y Cheng X Feng J . α-Lipoic acid alleviates myocardial injury and induces M2b macrophage polarization after myocardial infarction via HMGB1/NF-kB signaling pathway. Int Immunopharmacol. (2023) 121:110435. doi: 10.1016/j.intimp.2023.110435, 37320869

[75] Xie DM Zhong Q Xu X Li Y Chen S Li M . Alpha lipoic acid-loaded electrospun fibrous patch films protect heart in acute myocardial infarction mice by inhibiting oxidative stress. Int J Pharm. (2023) 632:122581. doi: 10.1016/j.ijpharm.2023.122581, 36608806

[76] Altunina NV Lizogub VG Bondarchuk OM. Alpha-lipoic acid as a means of influence on systemic inflammation in type 2 diabetes mellitus patients with prior myocardial infarction. J Med Life. (2020) 13:32–6. doi: 10.25122/jml-2020-0018, 32341698 PMC7175430

[77] Ozgun E Ozgun GS Usta U Eskiocak S Sut N Gokmen SS. The effect of lipoic acid in the prevention of myocardial infarction in diabetic rats. Bratisl Lek Listy. (2018) 119:664–9. doi: 10.4149/BLL_2018_119 30345777

[78] Wang X Song SM Lu WQ Zhao Y Lv RJ He Y . Alpha-lipoic acid alleviated intermittent hypoxia-induced myocardial injury in mice by promoting autophagy through Nrf2 signaling pathway. Eur J Pharmacol. (2025) 994:177380. doi: 10.1016/j.ejphar.2025.177380, 39954840

[79] Yang Z Tian Y Berr SS French BA. Therapeutic efficacy of alpha-lipoic acid against acute myocardial infarction and chronic left ventricular remodeling in mice. Cardiol Res Pract. (2020) 2020:6759808. doi: 10.1155/2020/6759808 32411448 PMC7199633

[80] Su Z Li P Ding W Gao Y. Urolithin A improves myocardial ischemia-reperfusion injury by attenuating oxidative stress and ferroptosis through Nrf2 pathway. Int Immunopharmacol. (2024) 143:113394. doi: 10.1016/j.intimp.2024.113394, 39437484

[81] Chen P Pei J Wang X Tai S Tang L Hu X. Gut bacterial metabolite Urolithin A inhibits myocardial fibrosis through activation of Nrf2 pathway *in vitro* and *in vivo*. Mol Med. (2022) 28:19. doi: 10.1186/s10020-022-00444-1, 35135471 PMC8822684

[82] Tang L Mo Y Li Y Zhong Y He S Zhang Y . Urolithin A alleviates myocardial ischemia/reperfusion injury via PI3K/Akt pathway. Biochem Biophys Res Commun. (2017) 486:774–80. doi: 10.1016/j.bbrc.2017.03.119, 28343995

[83] Yang Y Hu Q Kang H Li J Zhao X Zhu L . Urolithin A protects severe acute pancreatitis-associated acute cardiac injury by regulating mitochondrial fatty acid oxidative metabolism in cardiomyocytes. MedComm. (2023) 4:e459. doi: 10.1002/mco2.459, 38116065 PMC10728757

[84] Savi M Bocchi L Mena P Dall'Asta M Crozier A Brighenti F . *In vivo* administration of urolithin A and B prevents the occurrence of cardiac dysfunction in streptozotocin-induced diabetic rats. Cardiovasc Diabetol. (2017) 16:80. doi: 10.1186/s12933-017-0561-3, 28683791 PMC5501434

[85] Zhang Y Liu M Zhang Y Tian M Chen P Lan Y . Urolithin A alleviates acute kidney injury induced by renal ischemia reperfusion through the p62-Keap1-Nrf2 signaling pathway. Phytother Res. (2022) 36:984–95. doi: 10.1002/ptr.7370 35040204

[86] Xu MY Xu JJ Kang LJ Liu ZH Su MM Zhao WQ . Urolithin A promotes atherosclerotic plaque stability by limiting inflammation and hypercholesteremia in apolipoprotein E-deficient mice. Acta Pharmacol Sin. (2024) 45:2277–89. doi: 10.1038/s41401-024-01317-5, 38886550 PMC11489441

[87] Cui GH Chen WQ Shen ZY. Urolithin A shows anti-atherosclerotic activity via activation of class B scavenger receptor and activation of Nef2 signaling pathway. Pharmacol Rep. (2018) 70:519–24. doi: 10.1016/j.pharep.2017.04.020, 29660655

[88] Han QA Yan C Wang L Li G Xu Y Xia X. Urolithin A attenuates ox-LDL-induced endothelial dysfunction partly by modulating microRNA-27 and ERK/PPAR-γ pathway. Mol Nutr Food Res. (2016) 60:1933–43. doi: 10.1002/mnfr.201500827 27060359

[89] Lam-Sidun D. Effects of ergothioneine on endothelial cell and macrophage characteristics, and markers of atherosclerosis risk under high lipid conditions: the University of Western Ontario (Canada). (2021)

[90] Duan R Pan H Li D Liao S Han B. Ergothioneine improves myocardial remodeling and heart function after acute myocardial infarction via S-glutathionylation through the NF-ĸB dependent Wnt5a-sFlt-1 pathway. Eur J Pharmacol. (2023) 950:175759. doi: 10.1016/j.ejphar.2023.175759 37121564

[91] Cargnoni A Bernocchi P Ceconi C Curello S Ferrari R. *In vitro* administration of ergothioneine failed to protect isolated ischaemic and reperfused rabbit heart. Biochim Biophys Acta. (1995) 1270:173–8. doi: 10.1016/0925-4439(94)00084-4 7727541

[92] Liu Y Zhu W Ni D Zhou Z Gu JH Zhang W . Alpha lipoic acid antagonizes cytotoxicity of cobalt nanoparticles by inhibiting ferroptosis-like cell death. J Nanobiotechnol. (2020) 18:141. doi: 10.1186/s12951-020-00700-8, 33008409 PMC7532644

[93] Cheah IK Tang RMY Wang X Sachaphibulkij K Chong SY Lim LHK . Protection against doxorubicin-induced cardiotoxicity by ergothioneine. Antioxidants (Basel, Switzerland). (2023) 12:320. doi: 10.3390/antiox12020320 36829879 PMC9951880

[94] Huang JR Zhang MH Chen YJ Sun YL Gao ZM Li ZJ . Urolithin A ameliorates obesity-induced metabolic cardiomyopathy in mice via mitophagy activation. Acta Pharmacol Sin. (2023) 44:321–31. doi: 10.1038/s41401-022-00919-1, 35655094 PMC9889402

[95] Dos Santos SM Romeiro CFR Rodrigues CA Cerqueira ARL Monteiro MC. Mitochondrial dysfunction and alpha-lipoic acid: beneficial or harmful in Alzheimer's disease? Oxidative Med Cell Longev. (2019) 2019:8409329. doi: 10.1155/2019/8409329 PMC6914903 31885820

[96] Fong ZW Tang RMY Cheah IK Leow DMK Chen L Halliwell B. Ergothioneine and mitochondria: an important protective mechanism? Biochem Biophys Res Commun. (2024) 726:150269. doi: 10.1016/j.bbrc.2024.150269, 38909533

[97] Packer L Cadenas E. Lipoic acid: energy metabolism and redox regulation of transcription and cell signaling. J Clin Biochem Nutr. (2011) 48:26–32. doi: 10.3164/jcbn.11-005FR 21297908 PMC3022059

[98] Rahimlou M Asadi M Banaei Jahromi N Mansoori A. Alpha-lipoic acid (ALA) supplementation effect on glycemic and inflammatory biomarkers: a systematic review and meta- analysis. Clin Nutr ESPEN. (2019) 32:16–28. doi: 10.1016/j.clnesp.2019.03.015, 31221283

[99] Lam-Sidun D Peters KM Borradaile NM. Mushroom-derived medicine? Preclinical studies suggest potential benefits of Ergothioneine for Cardiometabolic health. Int J Mol Sci. (2021) 22:3246. doi: 10.3390/ijms22063246, 33806754 PMC8004618

